# Vaccine Inoculation Route Modulates Early Immunity and Consequently Antigen-Specific Immune Response

**DOI:** 10.3389/fimmu.2021.645210

**Published:** 2021-04-20

**Authors:** Pierre Rosenbaum, Nicolas Tchitchek, Candie Joly, André Rodriguez Pozo, Lev Stimmer, Sébastien Langlois, Hakim Hocini, Leslie Gosse, David Pejoski, Antonio Cosma, Anne-Sophie Beignon, Nathalie Dereuddre-Bosquet, Yves Levy, Roger Le Grand, Frédéric Martinon

**Affiliations:** ^1^ UMR1184 IMVA-HB, IDMIT Department, Université Paris-Saclay – INSERM U1184 – CEA, Fontenay-aux-Roses, France; ^2^ Vaccine Research Institute, Henri Mondor Hospital, Créteil, France; ^3^ INSERM, U1169, Kremlin-Bicêtre, France; ^4^ CEA – INSERM, MIRCen, UMS27, Fontenay-aux-Roses, France; ^5^ INSERM, U955, Team 16, Clinical and Infectious Diseases Department, Hospital Henri Mondor, University of Paris East, Créteil, France

**Keywords:** vaccine, innate & adaptive immune response, modified vaccinia Ankara (MVA), non human primates, administration routes, mass cytometry (CyTOF)

## Abstract

Vaccination is one of the most efficient public healthcare measures to fight infectious diseases. Nevertheless, the immune mechanisms induced *in vivo* by vaccination are still unclear. The route of administration, an important vaccination parameter, can substantially modify the quality of the response. How the route of administration affects the generation and profile of immune responses is of major interest. Here, we aimed to extensively characterize the profiles of the innate and adaptive response to vaccination induced after intradermal, subcutaneous, or intramuscular administration with a modified vaccinia virus Ankara model vaccine in non-human primates. The adaptive response following subcutaneous immunization was clearly different from that following intradermal or intramuscular immunization. The subcutaneous route induced a higher level of neutralizing antibodies than the intradermal and intramuscular vaccination routes. In contrast, polyfunctional CD8^+^ T-cell responses were preferentially induced after intradermal or intramuscular injection. We observed the same dichotomy when analyzing the early molecular and cellular immune events, highlighting the recruitment of cell populations, such as CD8^+^ T lymphocytes and myeloid-derived suppressive cells, and the activation of key immunomodulatory gene pathways. These results demonstrate that the quality of the vaccine response induced by an attenuated vaccine is shaped by early and subtle modifications of the innate immune response. In this immunization context, the route of administration must be tailored to the desired type of protective immune response. This will be achieved through systems vaccinology and mathematical modeling, which will be critical for predicting the efficacy of the vaccination route for personalized medicine.

## Introduction

Vaccination is one of the most effective public health approach to prevent infectious diseases. However, for many vaccines, the mechanisms responsible for suitable immunity are still poorly understood. In particular, vaccination routes are known to affect the quality of the vaccine response (1–3), although the underlying molecular and cellular mechanisms at play at the injection site and in the draining lymphoid tissues are yet to be elucidated. Better knowledge of the mechanisms triggered during the early response are needed to aid the design of more efficient vaccines.

Intramuscular (IM) injection is the most commonly used administration route, along with the subcutaneous (SC) route, due to their easy access and safety. However, muscle tissue has a low density of immune cells (4) and is thus not expected to be an optimal site for immunization (5). Therefore, the efficacy of the IM route requires a rapid diffusion of the vaccine antigens to draining lymph nodes (6). In contrast, skin tissue contains a broad array of antigen-presenting cells (APCs), which are likely to affect the magnitude, duration, and orientation of antigen-specific immune memory, and represents a potentially superior vaccine delivery site despite receiving less attention than classical administration routes due to practicality (7). Dose-sparing of up to ten times, without compromising immunogenicity, can be achieved *via* intradermal (ID) vaccination *versus* the SC and IM routes (8–10). In murine models, Mohanan et al. (11) showed that the administration route strongly influences the Th1 response, with higher IgG2a titers and IFNγ production favored by the IM and ID routes over the SC route.

Here, we determined how the administration routes can affect the magnitude and quality of the antigen specific memory response in cynomolgus macaques, used as an animal model to study human vaccines. Indeed, non-human primates (NHP) and human immune systems are highly similar, allowing the direct use of human vaccines in highly predictive NHP immunization models without the need of experimental adaptation, as well as many cross-reactive reagents (12, 13). We compared vaccine-induced innate and adaptive responses following administration *via* the SC, IM, and ID routes after prime (first immunization) and boost (a second injection two months after the prime), at the site of injection and at systemic level. We used the Modified Vaccinia virus Ankara (MVA), a living, attenuated, non-replicative vaccinia virus already safely used in humans as a 2^nd^ and 3^rd^ generation smallpox vaccine, as a model vaccine because of its capacity to elicit both strong B- and T-cell specific responses (14). This high immunogenicity combined with good safety has led MVA to be recently approved in Europe (market authorization EU/1/13/855/001) in case of malicious use of retained smallpox stocks for bioterrorism actions.

In addition, MVA is currently considered as a promising vector for new recombinant vaccine candidates against cancer and infectious diseases in humans (15–21). Our results show that the administration route of MVA strongly affects the quality and magnitude of the early innate response, and the subsequent installation of the MVA-specific response.

## Material and Methods

### Experiment Model and Subject Details

#### *In Vivo* Animal Studies

Thirty-five adult male cynomolgus macaques (*Macaca fascicularis*), each weighing between 4 and 6 kg, imported from Mauritius, were housed in the animal facilities of the CEA (Fontenay-aux-Roses, France). The macaques were handled in accordance with national regulations (Commissariat à l’Energie Atomique et aux Energies Alternatives – Permit Number A 92-32-02), in compliance with the Standards for the Human Care and Use of Laboratory Animals of the Office for Laboratory Animal Welfare (OLAW) under the Office for Laboratory Animal Welfare Assurance number A5826-01 and the European Directive (2010/63, recommendation N°9). This project received the authorization numbers 12-013 and 0201501281731916 (APAF1S#170).02 from the French Research Ministry, in accordance with French ethics policies. Data from animals used for adaptive response through subcutaneous route were shared in common with a related study (22). Interventions and follow-up of the animals (clinical examination, temperature, weight) were carried out by veterinarians and zootechnician staff of the “Animal Science and Welfare” core facility of the IDMIT infrastructure. Injections, tissue biopsies, and blood samples were performed after anesthesia with ketamine (10 mg/kg, Imalgen^®^, Rhone-Mérieux, Lyon, France) or tiletamin/zolazepam (5 mg/kg, Zolétil^®^, Virbac, Caros, France) for muscle biopsies.

#### Experimental Design

Three groups of four NHPs were included in the study of adaptive response. Randomization was performed based on their weight and MHC haplotypes. The experiments and data analysis were performed following protocols which were previously validated during in-house studies (23). Animals were immunized two times with MVA at a 58-day interval. Blood samples were collected at baseline and at days 8, 14, 28, and 57 post-prime and days 8, 14, 28, 57, and 104 (month 4) post-boost three additional groups of six NHPs, of equivalent average weight and MHC profile, were included to study the MVA-induced innate response. We evaluated the systemic response with blood sampling at baseline and at 3 h, 6 h, and 24 h after MVA immunization. An additional group of five cynomolgus macaques was used for studying the injection site after IM administration of MVA.

#### Immunization Procedures

The injection site was shaved and cleaned with 70% ethanol. Animals of the ID, SC, and IM groups simultaneously received injections containing a total of 4 x 10^8^ PFU of MVA expressing HIV proteins Gag, Pol, and Nef [MVA HIV-B MVATG17401 Transgene SA, Illkirch-Graffenstaden, France (24)] in 2 ml. For the study of IM injection site responses, 200 μl phosphate buffer saline (PBS, Gibco^®^) or recombinant MVA expressing eGFP [1 x 10^8^ PFU, MVATG15938, Transgene SA (25)] was injected into the biceps, triceps, or quadriceps muscles.

#### Tissue Biopsies

Prior to biopsies, the site of injection was cleaned with a povidone iodate solution (Vetedine^®^, Vetoquinol SA, Lure, France). Skin biopsies were 8 mm in diameter. For the subcutaneous route, 5 x 1.5 cm (ellipse shape) deep skin biopsies were performed with a scalpel. Muscular biopsies were conducted after anaesthesia of the animal with tiletamin/zolazepam (Zolétil^®^, 5 mg/kg). Targeted muscles were the triceps, biceps, and quadriceps. Biopsies were surgically removed at 24 or 48 h after injection.

#### Extraction of Cells From Tissue Biopsies

For biopsies after ID injection, subcutaneous tissue was removed to obtain only the epidermis and dermis, which were not separated afterwards. For biopsies after SC injection, all subcutaneous tissue was collected and the epidermis and dermis removed. Muscle biopsies after IM injection were cut into small pieces. All biopsy samples were then washed with PBS, cut into small pieces, weighed, and finally digested at 37°C under agitation for 60 min in 2 ml of a solution containing RPMI (Thermofischer Scientific), 5% FCS (Lonza, Basel, Switzerland), 1% penicillin/streptomycin/neomycin (Thermofischer Scientific), 10mM Hepes (Thermofischer Scientific), 2 mg/ml collagenase D (Roche, Basel, Switzerland), and 0.02 mg/ml DNAse I (Roche). Tissues were then filtered through a 70-µm cell strainer and centrifuged. The remaining skin sample was shredded with a GentleMACs^®^ dissociator (Miltenyi Biotec, Paris, France). The cell suspension was then washed with PBS and stained for flow cytometry analysis. Flow cytometry staining was performed from cell extractions coming from one single tissue biopsy.

#### Blood Sample Collection

Blood samples were collected into K3-EDTA tubes (Greiner Bio-One, Frickenhausen, Germany) for complete blood counts (CBC; HmX, Beckman Coulter), plasma, and flow cytometry analysis. For evaluation of anti-MVA binding antibody titers, serum was obtained from 2 mL of blood into serum clot activator tubes (Greiner Bio-One). Plasma and serum were collected after 10 min following centrifugation at 2000 x g. For transcriptomic analysis, a volume of 500 µL blood from Li-heparin tubes (Vacutainer BD) was added into a tempus RNA tube (Thermofischer Scientific, Waltham,MA, USA) containing 1 mL of buffer. For CyTOF staining, 10 mL of blood was collected in Li-heparin.

#### Measurement of Total Anti-MVA IgG and IgM in Serum

Experimental procedures were previously described (23). Briefly, wild type MVA (B. Verrier, Laboratoire de Biologie tissulaire et d’ingénierie thérapeutique, Institute of Biology and Chemistry of Proteins, Lyon, France) was used to coat 96-well MaxiSorp microplates (Nunc) at 10^5^ PFU/well in coating buffer (200 mM NaHCO_3_, 80 mM Na_2_CO_3_, pH 9.5) overnight at 4˚C. Wells were washed five times with wash buffer (PBS, 0.1% Tween 20, 10 mM EDTA) and blocked for 1 h at room temperature (RT) with 3% w/v BSA (Sigma). Plates were washed five times and incubated with two- or four-fold serial dilutions of macaque serum diluted in 1% w/v BSA in PBS for 2 h at RT, starting at 1:50 for IgG and 1:25 for IgM. Plates were then washed five times and peroxidase-conjugated goat anti-monkey H+L chain IgG (1:2,500) or IgM (1:1,000) (AbD Serotec) in 1% BSA (w/v) PBS was added and the plates incubated for 1 h at RT. Plates were washed five times, and 100 mL 3,3’,5,5’-tetramethylbenzidine (Thermo Scientific) added and the plates incubated for 30 min at RT in the dark. The reaction was stopped by adding 2N H_2_SO_4_. Absorbance was measured at 450 nm using a spectrophotometer (Multiskan FC, Thermo Scientific) and the data analyzed using SoftMax Pro software (version 4.6; Molecular Devices). Ab titers were calculated as described (23).

#### Antibody Neutralization Assay

Neutralization titers were determined using a modified version of a standard plaque inhibition assay, as previously described (23). Briefly, wild type MVA (1 PFU/cell) was mixed with an equal volume of four-fold serial dilutions of decomplemented serum in assay medium (DMEM, 2% FCS), starting at 1:20. After 60 min of incubation at 37°C, 0.1 ml of the serum-virus mixture was transferred, in duplicate, to a 96-well plate containing subconfluent UMNSAH/DF-1 chicken fibroblasts (ATCC). After 48 h of incubation at 37°C, cell viability was quantified using an MTS/PMS assay (CellTiter 96 AQueous Non-Radioactive Cell Proliferation Assay; Promega). Absorbance was measured at 492 nm using a spectrophotometer (Multiskan FC; Thermo Scientific) and the data analyzed using SoftMax Pro software (version 4.6; Molecular Devices). The sample dilution *versus* percentage viability was plotted (four-parameter logistic curve) to calculate the neutralizing concentration, corresponding to the sample dilution resulting in 50% neutralization of virus-mediated cell mortality. The viability of uninfected control cells and infected cells incubated with undiluted VIGIV (human polyclonal anti-vaccinia virus IgG; BEI Resources) was equivalent, as expected. Neutralization titers using VIGIV oscillated between 7 and 10 depending on the sample.

#### Flow-Cytometry Staining of Blood Samples

100 µL blood was stained for 30 min with 90 µL antibody mix in BD Horizon^®^ staining buffer (BD) containing the panel of antibodies listed in **Table S2** The red blood cells were removed with 1 mL BD FACs Lysing^®^ (BD) buffer for 10 min at RT and the remaining cells washed twice with PBS. The design of the cytometry panel and rationale for the gating strategies were mostly based on previous studies (26). We standardized the flow cytometry results based on complete blood count data to retain the absolute values in the blood, obtaining the number of cells per liter. The CBC-based number of cells per liter was calculated using the following formula: count obtained in flow cytometry of a given cell population multiplied by the CBC number of leukocytes divided by the count of total blood cells obtained by flow cytometry.

#### RNA Isolation and Microarray Sample Preparation

Biopsies were immediately immersed in RLT-beta-mercaptoethanol 1/100 lysis buffer (Qiagen, Courtaboeuf Cedex, France). Samples were then disrupted and homogenized using a TissueLyser LT (Qiagen, Courtaboeuf Cedex, France) and RNA purified using the Qiagen RNeasy Micro Kit, as previously described (23). Whole-blood RNA was recovered in tempus tubes (ThermoFisher scientific) and the RNA purified using the *Tempus*™ Spin RNA isolation kit (ThermoFisher Scientific). Contaminating DNA was removed using the RNA cleanup step of the RNeasy Micro Kit for both purifications. Purified RNA was then quantified using an ND-8000 spectrophotometer (NanoDrop Technologies, Fisher Scientific, Illkirch Cedex, France) before checking for integrity on a 2100 BioAnalyzer (Agilent Technologies, Massy Cedex, France). cDNA was synthesized and labeled with biotin using Ambion Illumina TotalPrep RNA Amplification Kits (Applied Biosystem/Ambion, Saint-Aubin, France). Labeled cRNA was hybridized to Illumina Human HT-12V4 BeadChips. All steps were performed following the manufacturer’s protocol.

#### Analysis of Transcriptomic Data

Functional-enrichment analysis of the various lists of differentially expressed genes was performed using Ingenuity Pathway Analysis software (IPA^®^, Qiagen, http://www.qiagen.com/ingenuity). Upstream regulators with a q-value < 10^-3^ and belonging to the cytokine-signaling, humoral immune-response, or pathogen-influenced signaling categories are represented with the exception of FSH, LH, and prolactin regulators. Canonical pathways with a q-value < 10^-3^ and belonging to the cellular immune-response, cytokine-signaling, humoral immune-response, or pathogen-influenced signaling categories are represented. Raw and normalized microarray data are available in the ArrayExpress database (27) under accession number E-MTAB-10309.

#### Multidimensional Scaling Representation

MDS representations were generated using the SVD-MDS algorithm (28). MDS methods aim to represent the similarities between high-dimensional objects, generally in two or three dimensions. The Kruskal stress (29) indicated at the bottom of the representation quantifies the quality of the MDS as the fraction of information lost during the dimensionality-reduction process. The MDS (30) representation in **Figures 2** and **3** was generated based on cell cluster profile calculated from SPADE analysis for each vaccination strategy. Each dot in the representation corresponds to one cluster from one administration route and distances between the dots are proportional to the cumulative difference of parameters between samples.

#### Linear Discriminant Analysis

LDA was performed based on cell-population abundances and transcript expression quantified in the blood for each vaccination strategy. The LDA (31) was parameterized to identify linear combinations of variables that best distinguish the immune response induced by the different administration routes.

#### Antibody Conjugation for CyTOF Staining

Pure (carrier-protein–free) mAbs or polyclonal Abs from various manufacturers were coupled to various elements using MAXPAR lanthanide labeling kits (Fluidigm, San Francisco, CA), as indicated in the manufacturer’s preload method for 400 µg Ab. The element–Ab combinations are shown in **Table S1**. The element–Ab conjugates were adjusted to 1 mg/ml in Ab stabilizer buffer (Candor Bioscience, Wangen, Germany), supplemented with sodium azide (Santa Cruz Biotechnology) to a final concentration of 0.01%, and stored at 4˚C under sterile conditions throughout the study. The element–Ab conjugates were titrated on PBMCs from healthy macaques in unstimulated and stimulated with PMA+ionomycin conditions to obtain optimal staining concentrations.

#### Intracellular Cytokine Staining of Blood PBMCs and CyTOF Staining

PBMC isolation was performed by overlaying a 1:1 (v/v) mixture of Li-heparin–collected whole blood and Dulbecco’s PBS (Invitrogen) on a mixture of 95% Lymphocyte Separation Media 1077 (GE Healthcare, PAA, Austria) and 5% PBS and centrifugation at 420 x g for 45 min at RT. PBMCs at the Separation Media–plasma interface were collected and washed twice in complete media (RPMI 1640 supplemented with 10% heat-inactivated FBS and 100 U/ml penicillin, streptomycin, and neomycin). Any remaining red blood cells were lysed for five minutes using a hypotonic solution and the pellet washed with PBS. Fresh PBMCs (5 x 10^6^/well) were then stimulated *in vitro* with wild-type MVA (MVA TGN33.1, Transgène) for 4 h and then incubated overnight at 37°C with brefeldin A (10 µg ml^−1^) (Sigma). The next day, 1 µL rhodium (Fluidigm) was mixed into each well. Cells were then incubated an additional 15 min at 37°C. Cells were then washed twice in staining buffer (SB; BD Biosciences, Franklin Lakes, NJ) and incubated for 45 min at 4°C with the element tagged surface-staining Abs (**Table S1**) in a total volume of 50 µl SB. Cells were washed twice with SB and then fixed in 2% paraformaldehyde (Electron Microscopy Sciences) in PBS for 45 min at 4°C. Cells were washed twice in SB, three times in permeabilization buffer (eBioscience), and then stained with intracellular Abs (**Table S1**) in 50 µl permeabilization buffer on ice for 30 min. Cells were washed twice with SB and resuspended in 0.25 mM iridium DNA intercalator (Fluidigm) in 200 µl PBS + 2% paraformaldehyde overnight at 4˚C, before washing once in SB, once in PBS, and three times in ddH_2_O, and filtered through a 5-ml polystyrene round-bottom tube with a 35 µm cell strainer cap (BD Biosciences). Fifty microliters of 4-Element EQ Beads (Fluidigm) were added to each sample, which were acquired as two replicates in a mass cytometer (CyTOF; Fluidigm) following the standard procedure recommended by the manufacturer (32).

#### Preprocessing of Mass-Cytometry Data

FCS files were normalized separately for each administration route experiment with the MATLAB Compiler software normalizer (33) using the signal from the 4-Element EQ beads (Fluidigm). Replicates were concatenated using R software. CD4^+^ and CD8^+^ T lymphocytes were manually gated using Cytobank^®^ software (34) as illustrated in **Figure S1**. Beads, doublets, and dead cells were removed. HLA‑DR^-^ and CD3^+^ cells were retained and considered to comprise mainly of T lymphocytes. CD4^+^ T cells were considered to be CD4^+^ and CD8^-^ whereas CD8^+^ T cells were considered to be CD4^-^ and CD8^+^. The isolated CD4^+^ and CD8^+^ T cells were then analyzed separately using automatic gating tools (30).

#### Identification of Cell Populations

The Spanning-tree Progression Analysis of Density-normalized Events (SPADE) algorithm was used to identify CD4 and CD8 T-cell populations with similar marker intensities. SPADE was performed using the publicly available R/Bioconductor package (35). Six separate SPADE analyses were performed, one for each of the following conditions: CD8 SC, CD8 ID, CD8 IM, CD4 SC, CD4 ID, and CD4 IM (**Figure S1**). For each SPADE analysis, we first randomly selected and concatenated an equal number of cells corresponding to the smallest sample uniformly for each sample to reduce bias during the SPADE down-sampling step caused by large variances between the numbers of cell events per macaque. We then performed the SPADE density-based down-sampling using a target of 30% of the preselected cells. SPADE analyses were based on 13 markers: Bcl2, GranzymeB, CD62L, CD69, CD154, CD197, IFNγ, IL2, IL17, Ki67, MIP1β, Perforin, and TNFα. These markers were selected because of their ability to define T-cell functions. The number of cell clusters chosen for the final analysis provided an optimal balance between phenotypically homogeneous clusters, which also contained adequate cell numbers for downstream analysis (22, 30).

The SPADEVizR (30) and CytoCompare (36) R packages were used to analyze the abundance and phenotypic similarity between the identified cell clusters. The phenotypic categorization of SPADE clusters in the heatmap representation was calculated using the 5^th^ and 95^th^ percentile range for each marker intensity and then dividing this range into five uniform categories. Categorization was computed based on the means of the individual SPADE expression medians for each marker. The categories represent negative, low, medium, high, and very high marker expression, using a color scale from white to dark red. The mean percentage of CD8^+^ or CD4^+^ T cells was computed for each SPADE cluster at each time point. Differentially abundant clusters (DACs) were identified using a two- sample t-test (p < 0.05) that compared the percentage of CD8^+^ and CD4^+^ T cells of a SPADE cluster with that at baseline at a given timepoint.

#### Gating Strategy of Blood Cells by Flow Cytometry and Analysis

The gating strategy is provided on **Figure S3A**. We optimized the panel using both NKG2a and CD33 markers on the same fluorochrome. Indeed, these markers are not expressed by the same cell populations (**Figure S3B**). First, doublets and debris were excluded. SSC-A^+^, CD66^+^ cells were considered to be granulocytes, which comprised mainly of neutrophils as demonstrated by the recruitment kinetic data derived from complete blood counts. However, we cannot exclude the presence of eosinophils and basophils in this gate. B lymphocytes were gated as Lin (CD3, CD20)^+^,CD4^-^, HLA-DR^+^ cells; CD8^+^ T lymphocytes as Lin (CD3, CD20)^+^, HLA-DR^-^, CD8^+^ cells; CD4^+^ T lymphocytes as Lin (CD3, CD20)^+^,CD4^-^, HLA-DR^-^ cells; and NK cells as Lin (CD3, CD20)^-^, CD16^+^, CD8^+^, NKG2a^+^ cells. Three main subtypes of monocytes were identified using CD14 and CD16 markers (37): non-classical monocytes as Lin (CD3, CD20)^-^, CD14^-^, CD16^+^; intermediate monocytes as Lin (CD3, CD20)^-^, CD14^+^, CD16^+^; and classical monocytes as Lin (CD3, CD20)^-^, CD14^+^, CD16^-^, HLA-DR^+^. pDCs were gated as Lin (CD3, CD20)^-^, CD14^-^, CD16^-^, HLA-DR^+^, CD123^+^ cells. Cells that were Lin (CD3, CD20)^-^, CD14^-^, CD16^-^, HLADR^mid^, CD123^+^ were considered to be remaining basophils, but could also contained HLADR^low^ pDCs. The CD33 marker allowed us to discriminate between two different DC subsets: Lin (CD3, CD20)^-^, CD14^-^, CD16^-^, HLA‑DR^+^, CD123^-^, CD33^+^ cells, named CD33^+^ DCs; and Lin (CD3, CD20)^-^, CD14^-^, CD16^-^, HLA‑DR^+^, CD123^-^, CD33^-^, named CD33^-^ DCs. In accordance with the literature (38), MDSCs were identified as follows: Lin (CD3, CD20)^-^, CD14^-^, CD16^-^, HLADR^-^, CD123^-^, CD66^low^, CD11b^+^, and most likely CD33^+^ for Lin^-^MDSCs and Lin (CD3, CD20)^-^, CD14^+^, HLADR^-^, CD11b^+^, CD33^+^ for CD14^+^ MDSCs. Cells expressing no markers in the panel were named “Remaining Cells”.

Flow cytometry data were scaled in the heatmap representations to have the same minimal and maximal expression values using R. Statistical analysis for the flow cytometry data was performed using Prism 6.0 (Graphpad Software Inc., La Jolla, CA, USA). Two-sided Friedman tests, followed by Dunn’s post-test were used to compare each timepoint with the baseline.

#### Immunohistochemistry

Samples were fixed at 4°C using 4% paraformaldehyde during 24h, then stored in PBS at 4°C. Paraffin inclusion was made using an automaton successively replacing PBS with alcohol, xylene, and paraffin. Tissues were cut afterward using microtome and set on slides. Hemalum Eosin coloration was then made using an automaton Microm HMS740 (Thermofischer Scientific), alternating xylene, alcohol, Hematoxylin (Labonord, Templemars, France), Eosin (VWR international, Fontenay-sous-Bois, France) baths.

#### Immunohistofluorescence

Skin and lymph-node biopsies were washed three times in PBS and then transferred into solution containing 14.6 g/L-lysine (Sigma Aldrich), 4% PFA (Sigma Aldrich), and 2 g/L sodium(meta)periodate (Sigma Aldrich) in 0.05 mol/l phosphate Buffer (made using 1 mol/L monobasic and dibasic potassium phosphate solutions in equal quantities (Sigma Aldrich) at 4°C for 8 h. Then, skin samples were washed in PBS and transferred to a 30% sucrose (Sigma-Aldrich) solution and incubated overnight. Tissues were then frozen embedded in optimal cutting temperature compound (OCT) in dry ice and acetone. Staining was performed according to the following steps (rinsing with PBS between steps). First, cells were permeabilized for 30 min at 37°C in PBS containing 0.3% Triton X-100 (Sigma Aldrich), followed by a saturation step for 30 min at 37°C in PBS containing 10% BSA (Sigma Aldrich) and 10% Macaque serum (In-lab production). Next, primary antibody staining was performed for 2 h at 37°C in 100 µl PBS with 0.2% BSA containing 1.5 µl of anti-E3L (NIH Biodefense and Emerging Infections Research Resources Repository, NIAID, NIH: Monoclonal Anti-Vaccinia Virus E3L, Clone TW2.3 (produced *in vitro*), NR-4547.), 0.5 µl of anti-CD66 (clone TET2, Miltenyi Biotec), and 6 µl of anti-CD163 (clone GHI/61, Biolegend). Finally, slices were stained for 1 h at 37°C with secondary antibodies (Thermofischer Scientific). A post-fixation step in 4% PFA (15 min) was then performed, followed by DAPI staining (Thermofischer Scientific), and the samples mounted. Stained slides were then conserved at 4°C.

#### Quantification and Statistical Analysis

**Figure 1:**
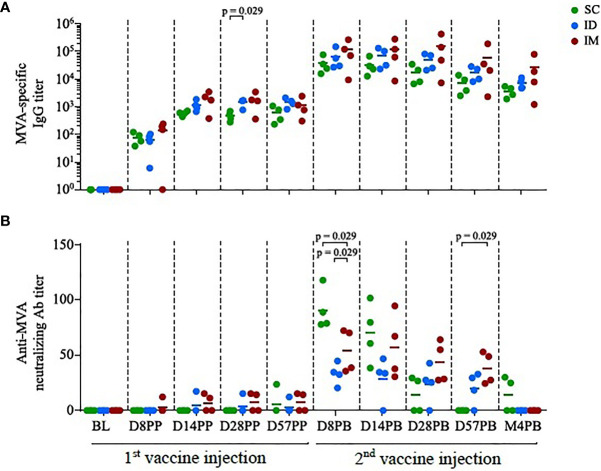
Antibody responses induced by homologous MVA prime-boost administered by the SC/ID/IM routes. **(A)** Scatter plot of MVA-specific IgG titers over time, characterized by ELISA. Each dot corresponds to one single biological sample. The colors correspond to the different administration routes. **(B)** Scatter plot of MVA neutralization titers over time, characterized by plaque reduction assay. Each dot corresponds to one single biological sample. BL, baseline; D, day; PP, post-prime; PB, post-boost; M, month.

**Figure 2:**
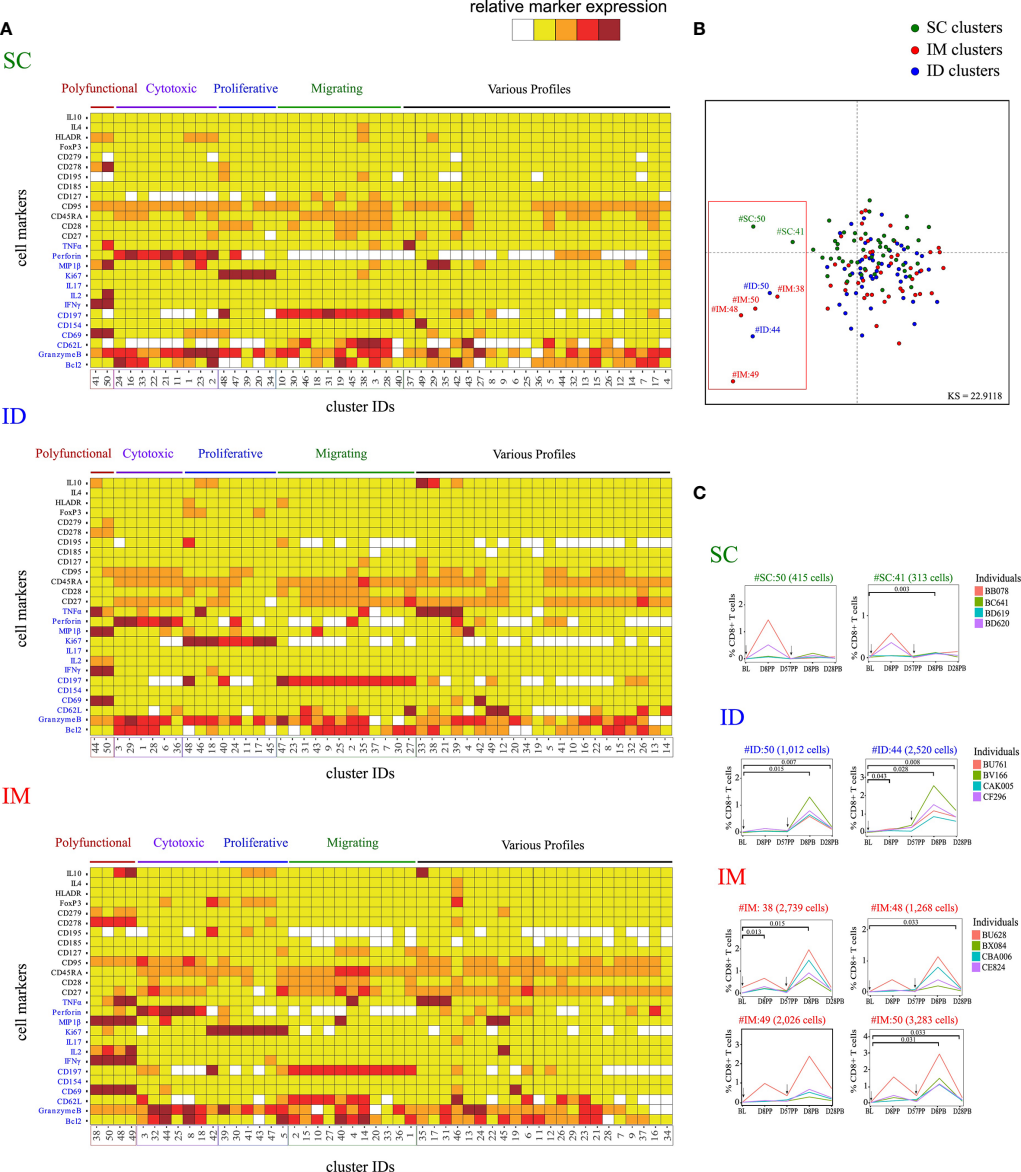
Characterization of the impact of the SC/ID/IM routes on CD8^+^ T-cell phenotypic cluster diversity by mass cytometry. **(A)** Categorical marker expression heatmap representing the phenotypes of CD8^+^ T cells for each administration route. SPADE analysis was performed for each administration route based on the entire cell set according to the gating strategy described in **Figure S1**. Markers in blue were used as clustering markers in the SPADE analyses. Cell clusters were organized into families by hierarchical clustering. The five-tiered color scale, from white to deep red, represents relative marker expression. **(B)** Two-dimensional MDS representation showing the phenotypic differences and similarities between cell clusters identified by each SPADE analysis. Phenotypic similarities were quantified based on the mean signal intensity of the 13 SPADE clustering markers. Red square indicate the eight singular cell cluster **(C)** Charts showing changes in the percentage of CD8^+^ T cells over time for the selected marginal clusters. Black arrows indicate the days of vaccine injection. BL, baseline; D, day; PP, post-prime; PB, post-boost.

**Figure 3:**
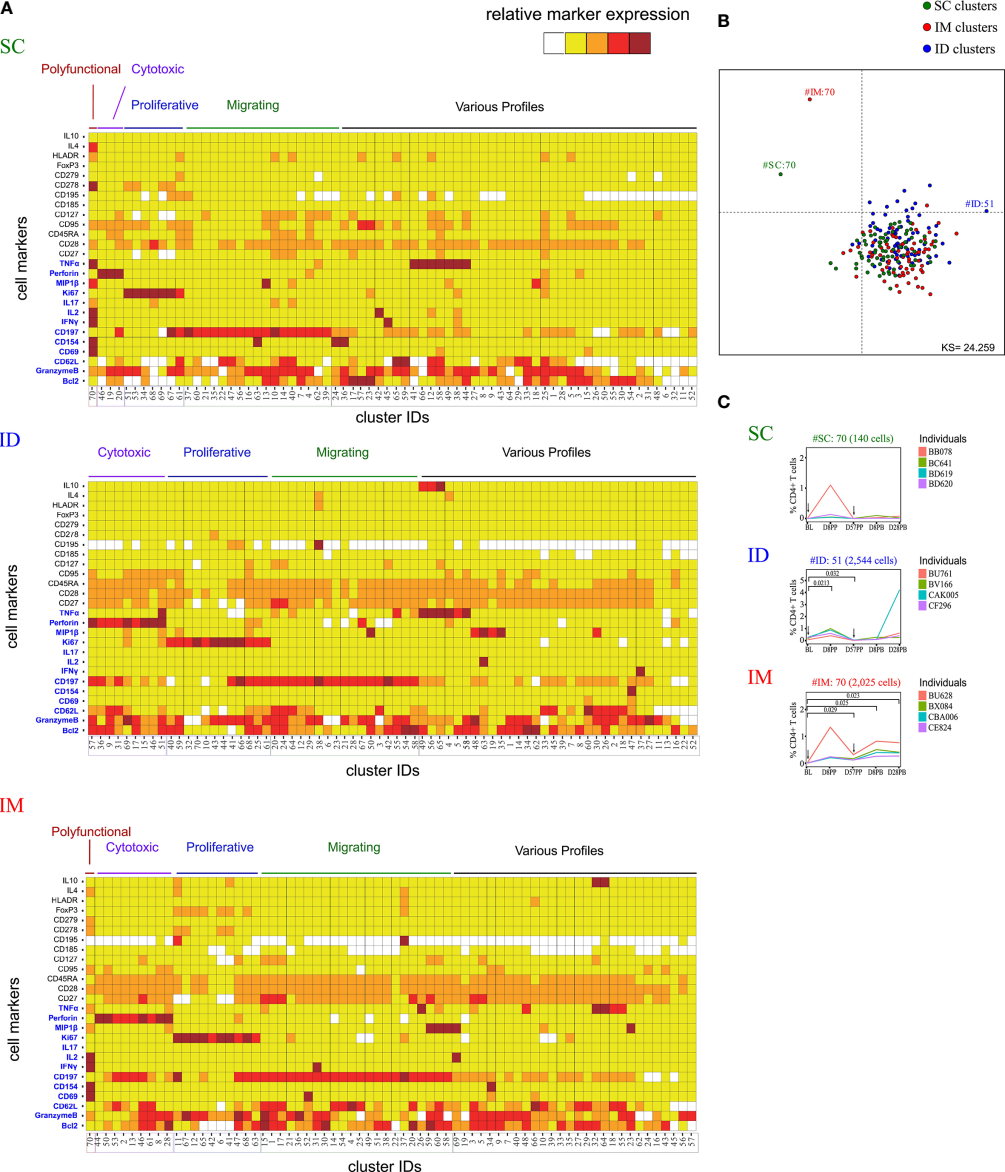
Characterization of the impact of the SC/ID/IM routes on CD4^+^ T-cell cluster diversity by mass cytometry. **(A)** Categorical marker expression heatmap representing the phenotypes of CD4^+^ T cells for each administration route. SPADE analysis was performed for each administration route based on the entire cell set according to the gating strategy described in **Figure S1**. Markers in blue were used as clustering markers in the SPADE analyses. Cell clusters were organized into families by hierarchical clustering. The five-tiered color scale, from white to deep red, represents relative marker expression. **(B)** Two-dimensional MDS representation showing the phenotypic differences and similarities between cell clusters identified by each SPADE analysis. Phenotypic similarities were quantified based on the MSI of the 13 SPADE clustering markers **(C)** Charts showing changes in the percentage of CD4^+^ T cells over time for the selected marginal clusters. Black arrows indicate the days of vaccine injection. BL, baseline; D, day; PP, post-prime; PB, post-boost.

**Figure 4:**
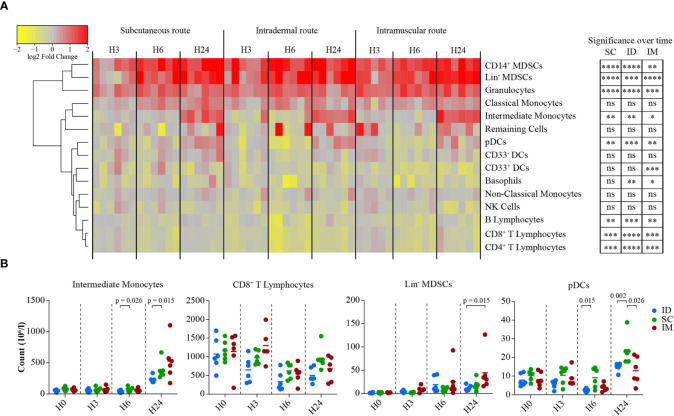
Impact of the SC/ID/IM routes on early systemic immunity. **(A)** Heatmap representation showing the changes in cell abundance (in log2 fold change) of populations characterized by flow cytometry in the blood 3, 6, and 24 h after SC/ID/IM MVA injection. Populations were organized based on their cell abundance by hierarchical clustering and are represented by a dendrogram. Changes in cell abundance of each population were normalized and are represented by a color-gradient scale ranging from yellow to red. Significant changes over time are indicated on the right **(B)** Scatter plot showing the kinetics of changes in cell abundance for selected cell populations. Each dot represents one single biological sample. ns, non-significant; *0.05 > p > 0.01; **0.01 > p > 0.001; ***0.001 > p > 0.0001; ****p < 0.0001.

**Figure 5:**
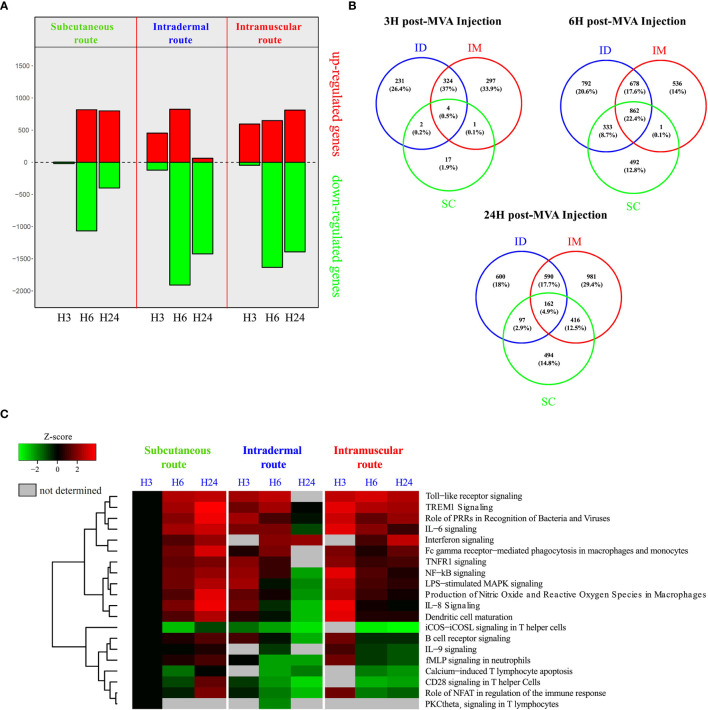
Transcriptomic analysis of systemic early responses induced by SC/ID/IM MVA administration. **(A)** Histogram of the number of significantly up-and downregulated genes at 3, 6, and 24 after MVA injection in the whole blood relative to baseline (0 h) for each administration route. Downregulated genes are represented in green and upregulated genes in red. **(B)** Venn diagrams showing the overlaps between the differentially expressed genes for each administration route at 3, 6, and 24 h after MVA injection. **(C)** Heatmap representation showing functional enrichment scores for significantly over-represented canonical pathways related to immune processing and cytokine signaling with a p-value < 10^-3^. The color scale corresponds to the Z-score, which is proportional to the upregulation (in red) or downregulation (in green) of the pathways.

**Figure 6:**
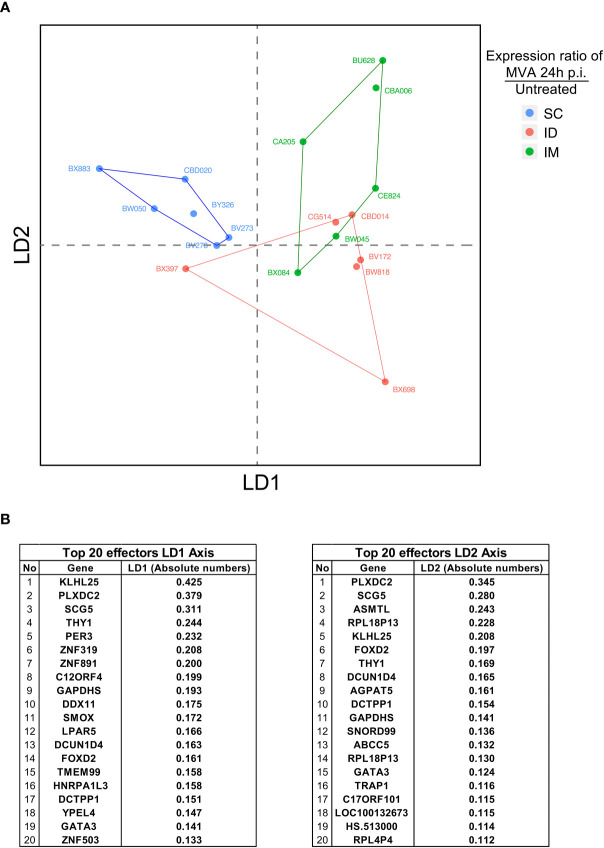
Integrative analysis of innate immune signatures after ID/IM/SC MVA immunization. **(A)** LDA representation of the innate dataset after ID, IM, and SC MVA immunization. Each dot corresponds to one biological sample. Each color corresponds to one administration route. Biological samples are represented on a two-dimension scale. Differential gene expression between samples was translated into the Euclidian distance between samples. **(B)** Top 20 identified genes involved in the level of expression of samples on the LD1 axis and the LD2 axis.

**Figure 7:**
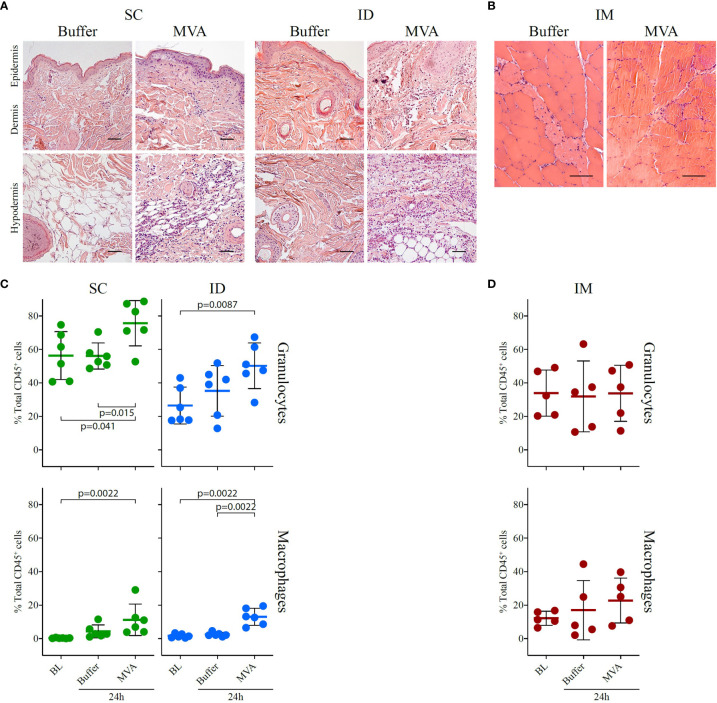
Local inflammatory response 24 h after MVA immunization by the SC or ID route. **(A)** HE staining of the injection site (skin + subcutaneous tissue) and draining inguinal lymph node, showing transversal sections of paraffin-embedded tissue cassettes 24 h after ID or SC injection of buffer or rMVA. The scale bar is equal to 50 µm. One representative experiment of two is shown. A scatter plot representation of local cellular movement to the site of injection at baseline and following injection with buffer or MVA by the SC or ID route is shown at the bottom. **(B)** HE staining of muscle, showing transversal sections of paraffin-embedded tissue cassettes at baseline and 24 h after injection with PBS and 24 h after MVA immunization. The scale bar is equal to 50 µm. On the bottom, scatter plot representation of local cellular movement to the site of injection at baseline and after injection with buffer or MVA after **(C)** SC or ID route and **(D)** IM route. Each dot represents one biological sample (n = 6 for the ID and SC groups and n = 5 for the IM group). Values correspond to the percentage of cells among CD45^+^ cells conforming to the gating strategy in **Figure S5A** for the SC and ID group and **Figure S5B** for the IM group. Statistical analysis consisted of a Mann-Whitney test test.

Statistical analysis for the **Figures 1**, **4** and **7** were performed using Prism 6.0 (Graphpad Software Inc., La Jolla, CA, USA). Statistical analysis for the **Figures 2**, **3**, **6**, and **Figure S2** were performed using R software. Statistical analysis for **Figure 5** was determined by Ingenuity Pathway analysis Software (Qiagen Inc. Hilden, Germany).

**Figure 1**: Four subjects per group. The bar represents the mean. Statistical significances was determined by Mann-Whitney test

**Figure 2**/**Figure 3**/**Figure S2**: Four subjects per group. Statistical significances was determined by comparing each group by t-test

**Figure 4A**: Six subjects per group.Two-sided Friedman tests, followed by Dunn’s post-test.

**Figure 4B**: Six subjects per group. The bar represents the mean. Statistical significances was determined by Mann-Whitney test

**Figure 5**: Six subjects per group.

**Figure 6**: Six subjects per group. The bar represents the mean. Statistical significances was determined by Mann-Whitney test

## Results

### The Route of Immunization Induced Distinct Profiles of Neutralizing Antibodies in Serum

Three groups of four macaques were immunized two times with 4 x 10^8^ PFU rMVA, injected ID, SC, or IM at 58-days intervals. MVA-specific IgG have similar kinetic profiles in all groups. The impact of administration route on antibody titers appeared to be limited (**Figure 1A**), although significant at day 28 post-prime (D28PP) (SC vs ID, p = 0.029). A trend to maintain higher responses four months post-boost (M4PB) was also observed after IM route. In contrast, the SC route was significantly more efficient in inducing MVA-neutralizing activity early after the boost than the ID (p = 0.029 at day 8 post-boost (D8PB); p = 0.057 at day 14 post-boost (D14PB); or IM routes (p = 0.029 at D8PB) (**Figure 1B**). Interestingly, IM immunization appears to induce lower and delayed peaks of serum neutralizing responses, becoming significantly higher when compared to SC by day 28 post-boost (p = 0.029 at D28PB and D57PB). However, these neutralizing activities did not persist over time. These changes in profiles of responses in spite of comparable binding IgG levels suggest that paths of antigen uptake, processing or presentation, may be driven by distinct inflammatory processes and populations of innate cells, accordingly to injections sites in a way that could modulate the quality (IgG isotypes, IgM and IgA, MVA recognized epitopes) of the specific antibodies and associated functionalities.

### MVA Immunization Through the ID/IM Route Favors Polyfunctional Specific CD8^+^ T Cells

The MVA-specific CD8^+^ T-cell responses were characterized by the intracellular production of cytokines following PBMC stimulation *ex vivo* with MVA for 18 h. Assays were performed over the kinetics of immunization at baseline (BL), D8PP, D28PP, D8PB, and D28PB. Cells were stained with a panel of 29 antibodies coupled to heavy metals (**Table S1**) and the data acquired by mass cytometry. The SPADE algorithm (39, 40) was used on gated CD8^+^ T cells to identify cell clusters based on the expression of 13 functional and homing markers; Bcl2, Granzyme B, CD62L, CD69, CD154, CD197, IFNγ, IL-2, IL-17, Ki67, MIP1β, Perforin, and TNFα (**Figure S1**). Due to different experiment timeframe and different antibody batches, one SPADE analysis per administration route was performed. Each SPADE analysis was performed using data from all animals and timepoints. Each SPADE contained 50 clusters. Phenotypes (**Figure 2**) and cell abundances (**Figure S2**) of each cluster were represented using categorical heatmaps based on the relative range of expression of each marker (five categories from no expression to very high expression). Hierarchical clustering at the cell cluster level was used to define groups of clusters with closely related phenotypes, which were designated as ‘phenotypic families’. We identified five phenotypic families. Clusters of polyfunctional cells expressed a backbone of markers related to T-cell activation, including CD69 and IFNγ, and high levels of MIP1β, TNFα, and IL2. In addition, other key markers were heterogeneously expressed by these polyfunctional clusters, such as IL10 (expressed in two of four polyfunctional clusters of the IM route group) and CD278 (expressed moderately in the SC and strongly in the IM group). The phenotypic family with cytotoxic activity markers was defined by the expression of perforin and Granzyme B. The proliferative family was defined by the expression of Ki67 and the migrating family by the expression of homing receptors to secondary lymphoid organs, such as CD197 and CD62L. Finally, the remaining cell clusters were classified into phenotypic family named “various profiles”. Using multidimensional scaling (MDS) analysis (**Figure 2B**), we identified eight clusters with singular phenotypes all part of the polyfunctional families. Cells associated with SC-specific cell clusters with the polyfunctional phenotypes SC#50 (415 cells) and SC#41 (313 cells, p = 0.003 at D8PB) appeared to increase in only two macaques (**Figure 2C**) and at low magnitude. In contrast, abundance of the two polyfunctional clusters associated with ID route (clusters ID#44 and ID#50) mainly increased at D8PB (ID#44, p = 0.043) at D8PP (day 8 post-prime), but were differentially abundant at D8PB (ID#44, p = 0.028; ID#50, p = 0.015) and day D28PB (ID#44, p = 0.008; ID#50, p = 0.007) relative to the baseline. The IM route induced four polyfunctional clusters (IM#38, IM#48, IM#49, and IM#50), which also displayed changed kinetic profiles similar to the ID route, with significant increases at D8PB (IM#38, p = 0.015; IM#50, p = 0.032) and D28PB (IM#50, p = 0.033). In addition, the IM route induced more heterogeneous marker expression. In particular, IL-10 was only expressed in two of four polyfunctional clusters of the IM route.

CD8^+^ T cells of the cytotoxic group were not particularly more enriched after vaccination by the SC or IM route, whereas two clusters were more enriched at D8PB after vaccination by the ID route (**Figure S2A**). Clusters from the migrating-cells phenotypic family also became more abundant after immunization *via* the ID route. These results highlight that the MVA administration route alters the CD8^+^ response profile. In particular, IFNγ-secreting CD8^+^ T cells displayed a distinct polyfunctional profile and followed different recruitment kinetics. Vaccination by the IM route induced a significant increase in the number of antigen-specific polyfunctional CD8^+^ T cells associated with increased expression of other markers, including IL10. In contrast, vaccination by the SC route induced limited MVA-specific CD8^+^ T-cell responses. Vaccination by the ID route appeared to induce an intermediate level of responses, with a moderate increase in polyfunctional activity, but also a cluster abundance increase in cytotoxic and migratory phenotypic families in comparison with other immunization routes. We surmise from these results that early molecular and cellular changes at the site of antigen exposure may significantly impact on the initiation of antigen specific CD8+ T cell responses.

### The CD4+ T-Cell Response Profile Is Affected by the Administration Route of MVA

Differences in MVA specific antibodies and CD8+ T cells functionalities and profiles may be associated to differences in CD4^+^ T cell compartment accordingly to immunization route. The characterization of CD4^+^ T cells was performed similarly to that of the CD8^+^ T cells (**Figure S1**), with the same antibody panel (**Table S1**). Three SPADE analyses were performed to identify 70 clusters for each administration route, and the phenotypes (**Figure 3A**) and cell abundances (**Figure S2B**) of each cell cluster were analyzed. Five phenotypic families were defined for each SPADE analysis based on marker expression, similarly to that of the CD8^+^ T cells.

MDS analysis identified three phenotypically distinct clusters (**Figure 3B**): one specific to the SC route (SC#70), one specific to the IM route (IM#70), and one specific to the ID route (ID#51). Two harbored a polyfunctional phenotype (SC#70 and IM#70) and expressed CD154 in addition to CD69, IFNγ, TNFα, and IL-2, corresponding to a Th1 profile. In contrast, the ID-associated cluster (ID#51), expressing high levels of TNFα, was classified in the cytotoxic family because of the high expression of perforin and granzyme B. The kinetics of cell abundances within the clusters showed that the SC-specific cluster (SC#70) corresponded to cells originating mostly from one animal at D8PB (**Figure 3C**). However, the IM-specific cluster (IM#70) had significantly more cells in all animals at D57PP (p = 0.029), D8PB (p = 0.025), and D28PB (p = 0.023) relative to baseline. The ID-specific cluster (ID#51) showed differential abundance at D8PP (p = 0.021) and D57PP (p = 0.032) only.

The abundance of cytotoxic clusters expressing high levels of perforin and granzyme B significantly increased over time following SC administration, in contrast to IM or ID administration (**Figure S2B**). MVA induced an increase of CD4^+^ T cells exhibiting proliferation markers at D8PP in each administration route. This increase was however transient in the IM and ID groups.

Overall, the CD4^+^ T-cell response was affected by the administration route, similarly to that of CD8^+^ T cells. A marked Th1 signature following IM and ID immunization, whereas the SC route induced a weaker Th1 response, with a lower abundance of polyfunctional cluster cells.

### MVA-Induced Systemic Inflammation Echoes the Adaptive Response

Since we observed that adaptive immune profiles were largely polarized depending on the administration route of the MVA, we hypothesized that it was shaped by the early innate response following vaccine injection.

We performed flow cytometry analysis (**Figure S3**) at 3, 6, and 24 h after MVA injection in additional groups of six NHPs to decipher the MVA-induced early innate immune cell changes in the blood compartment (**Figure 4**). Myeloid cells populations fluctuated globally similarly for the three routes, with a peak reached after 6 and 24 h post-MVA injection. The three routes of immunization induced significant recruitment of granulocytes (p < 0.0001 for SC, ID, and IM) and intermediate monocytes (p < 0.01 for SC and ID and p = 0.05 for IM) over time, but with distinct frequencies accordingly to injection site. Intermediate monocytes (SC *vs*. ID after 6 h: p = 0.026; SC *vs.* ID after 24 h: p = 0.015), CD8^+^ T cells (SC *vs.* ID after 24 h: p = 0.0087) and pDCs (SC *vs*. ID after 6 h: p = 0.015; SC *vs.* ID after 24 h: p = 0.0022; SC *vs.* IM after 24 h: p = 0.026) were significantly less recruited after vaccination by the ID and/or IM routes compared to the SC route.

Interestingly, these changes were associated to a significant increase in the number of myeloid-derived suppressive phenotype (MDSCs), including CD14^+^ MDSCs (p < 0.0001 for SC and ID and p = 0.005 for IM), as well as Lin^-^ MDSCs (p < 0.0001 for SC and IM and p = 0.005 for ID) (**Figure 4A**). This suggested that these cells play a physiological role in controlling early inflammation. A greater recruitment of Lin^-^MDSCs following vaccination by the IM route was observed compared to the ID route (ID *vs.* IM after 24 h: p = 0.015 after 24 h) (**Figure 4B**). Conversely, B lymphocytes (p < 0.01 for SC and IM and p = 0.05 for ID), CD8^+^ T lymphocytes (p < 0.001 for SC and IM and p = 0.0001 for ID), and CD4^+^ T lymphocytes (p < 0.001 for SC and IM and p = 0.0001 for ID) were reduced in the blood compartment between 6 and 24 h post-MVA injection (**Figure 4B**). As a result, there were subtle but clear differences in the early kinetics of key immune cell subsets observed. The response following the SC route differed from that of the ID and/or IM routes for three of the four cell populations: CD8^-^ T cells, intermediate monocytes, and CD14^+^ MDSCs.

### The Early Kinetics of Gene Expression Signatures Distinguishes the SC From the ID/IM Route

To complement characterization of cell changes, we additionally studied the early immune mechanisms following vaccination at the molecular level by performing transcriptomic profiling of blood samples at 3, 6, and 24 h after MVA immunization (**Figure 5**). Gene expression analysis highlighted different kinetic patterns, depending on the administration route (**Figure 5A**). Indeed, more mRNA expression appeared to be decreased after vaccination by the IM and ID route than the SC route. The kinetics of increased genes products differed between groups, despite a comparable magnitude (peak of approximately 800 increased transcripts). Furthermore, the peak for the SC route was observed after 6 h and persisted until 24 h, whereas the peak for the ID route was transient, reaching a maximum peaking at 6 h and then rapidly decreasing by 24 h. Following IM injection, the number of increased gene products was already high after 3 h and continued to increase up to 24 h.

Gene expression analysis showed that 22.4% of the differentially enriched transcripts were common between the three routes at 6 h (**Figure 5B**). Notably, more transcripts were in common between the IM and ID routes (37% at 3 h, 17.6% at 6 h, and 17.7% at 24 h) than between the ID and SC routes (0.2% at 3 h, 8.7% at 6 h, and 2.9% at 24 h) and the IM and SC routes (0.1% at 3 h, 0.1% at 6 h, and 12.5% at 24 h).

Functional enrichment analysis showed that the modulation of several immune-related pathways were shared between all three conditions (**Figure 5C**), such as innate functions associated with lL-6 and TREM1-signaling. Most of the significantly immune-related increased pathways reached a peak at 3 h after vaccination by the IM and ID routes. Conversely, the same pathways plateaued 24 h after vaccination by the SC route. The magnitude of the pathways associated with the innate immune response was globally less, especially for the ID route (TREM1 signaling Z-score: z = 3.87 for SC, 3.46 for IM, and 2.5 for ID at the peak of the response). This is illustrated by a decrease of the inflammation-related pathways at 24 h, such as LPS-stimulated MAPK signaling, dendritic-cell (DC) maturation, and IL-8 signaling. In addition, several pathways related to the lymphoid response (such as iCOS-iCOSL signaling in T helper cells, calcium-induced T lymphocyte apoptosis, and CD28 signaling in T helper cells) were decreased after vaccination by the IM and ID route but not the SC route.

Overall, transcriptomic profiling varied depending on the administration route. However, because of rapid and significant changes occurring in cell subtypes proportions it is difficult to discriminate the mRNA variations reflecting these changes from true gene expression modulations. However, canonical pathways associated with pathogen sensing, proinflammatory responses, and T cell regulation also followed different kinetics and differed in magnitude depending on the administration route. Dynamics of gene expression into these pathways categorized the SC group (peak at 24 h) separately to the IM and ID groups (peak at 3 h). This may be consistent with differences observed for antibody and CD8^+^ T cell responses induced, on the one hand, by IM and ID routes and, on the other hand, by SC route.

### Integrative Analysis of the Innate Response Profile Suggests That Early Innate Immune Effectors Drive the Adaptive-Response Profile

We performed an integrative analysis of all innate immune response data to evaluate the specificity of the routes. We used the data of cell populations identified by flow cytometry and differentially enriched gene products after 24 h post-injection, which corresponds to the peak in terms of magnitude of cellular and transcriptomic signatures (**Table S3**).

A linear discriminant analysis (LDA) was used to compare the different routes (**Figure 6**). LDA aims to identify linear combinations of variables (named Linear Discriminant) that best segregate samples from the different conditions. This approach allowed us to confirm the dichotomy between the IM/ID conditions relative to the SC condition, mainly driven by LD1 (**Figure 6A**). The important imbalance in the number of parameters integrated for each type of data (low numbers of variables for flow cytometry in comparison with transcriptomics) bias the analysis and certainly explain that the main factors affecting this segregation were changes in transcripts enrichment rather than immune cells populations. Furthermore, values of the top 20 factors were quite homogeneous, suggesting a comparable contribution of these factors to drive SC specificity (**Figure 6B**). These factors included genes encoding proteins with very different functions. Some of them depicted a strong contribution but were not directly related to immune function, such as PLXDC2 (plexin domain containing 2, involved in the inhibition of angiogenesis) (41), SG5 (encoding a chaperone protein of a neuroendocrine enzyme) (42) and PER3 (involved in the circadian rhythm). Other factors were associated with innate immune processes, such as KLHL25 (associated with MHC I presentation) and TRAP1 (TNF response). Some of the identified genes were associated with T-cell maturation and regulation, such as Thy-1 (43) and GATA-3. Overall, the segregation profiles were more the sum of slightly modified signatures than the contribution of a few genes with specific immune functions.

In conclusion, this integrative analysis of the blood innate immune response showed that early signatures induced by the IM and ID route differ from those induced by the SC route. Mechanisms associated with SC segregation appear to belong to various signaling pathways. These analyses of the innate immune response support the hypothesis that the early innate immune profile can shape the adaptive response. Furthermore, these findings underlined the importance of investigating holistic analytical approaches. Indeed, in this study, parameters that were not known to be primarily involved in immune process could also be a part of an early signature that is associated with alteration of MVA-specific T cell and antibody responses.

### Innate Response at the Site of Injection

A comprehensive understanding of the initiation of MVA vaccine-induced responses requires the characterization of molecular and cellular changes in tissues at site of injection, which may not be fully reflected by whole blood analysis. We thus compared granulocyte and macrophage recruitment between baseline, buffer immunization, and 24 h after MVA immunization by histology and flow cytometry. Due to technical constraints, the analysis after vaccination by the IM route was performed under different conditions than that by the SC and ID route, detailed in Materials and Methods.

The analysis of tissue slides by histology revealed that ID MVA immunization induced inflammatory cell infiltration, infiltrating mostly subcutaneous tissue, as well as the dermis after 24 h (**Figure 7A**). SC MVA immunization induced similar local infiltration, mostly located in the subcutaneous tissue (**Figure 7A**). There was no major local inflammation after IM MVA immunization (**Figure 7B**). Looking more precisely the nature of cell populations with flow cytometry, MVA immunization induced clear local recruitment of granulocytes for both the SC and ID routes (SC route baseline *vs.* MVA 24 h: p = 0.041, buffer 24 h *vs.* MVA 24 h: p = 0.015, and ID route baseline *vs.* MVA 24 h: p = 0.0087), as well as macrophages (SC route baseline *vs.* MVA 24 h: p = 0.0022; ID route baseline *vs.* MVA 24 h: p = 0.0087, and buffer 24 h *vs*. MVA 24 h: p = 0.0022) (**Figure 7C**). Although the exact localization of the injection was difficult to determine in muscle biopsies, we detected a trend for increased recruitment of macrophages characterized after IM immunization (**Figure 7D**). As a result, we observed an increase of granulocytes and/or macrophages 24h after any route of vaccine immunization. However, the localization and the amplitude of this response differed depending on the route. Overall, similarly to innate systemic signature, we observed shared phenomenon but also discrepancies in the local inflammatory response after SC, ID, or IM MVA administration that may be reflected in the blood.

## Discussion

Administration route can shape immune response profiles after vaccination (44, 45). Yet, crucial players of this innate and adaptive immune response remain to be identified. Here, we investigated the effect of the commonly used SC and IM in addition to ID administration routes on the stimulation of immune responses in an MVA vector-based, live attenuated NHP vaccine model. We combined a complete set of experimental data obtained by multiparameter techniques, at the cellular (flow cytometry and mass cytometry) and molecular (transcriptomics) levels, with systems vaccinology approaches, providing a holistic view of the complex mechanisms of the vaccine response, to evaluate the impact of the administration route on the immune response.

MVA administration induced substantial myeloid cell recruitment, whereas B and T lymphocytes were reduced in the blood compartment. Although the cell trafficking was globally similar, in-depth analysis revealed that the early immune response shows route-specific differences. For instance, CD8^+^ T lymphocytes and pDCs, expanded more in between 6 to 24 h after SC immunization compared to after the IM and ID routes. In addition, the functional enrichment of differentially expressed genes was strongly associated with immune pathways peaking at 24 h for the SC route and 3 h post-injection for the IM and ID routes. These pathways were mostly associated with acute responses, *i.e.* IL-6, nitric oxide production, and triggering of receptors expressed on myeloid cells (46, 47). An integrative analysis based on the whole set of innate immune parameters showed the same polarization between the IM/ID and SC routes. These results reinforce the study of Gonçalves et al. with an inactivated influenza vaccine showing that gene signatures from the early innate immune response can be predictive of adaptive immune profiles (48). In this work with an attenuated virus model, administration routes also appear to orientate the ensuing immune responses, which contribute to the body of knowledge describing the effects of innate immunity on adaptive immunity.

MVA administration through the IM and ID routes was associated with a Th1-oriented profile, with an increase in MVA-specific CD8^+^ T lymphocytes producing IFNγ, TNFα, MIP1β, and IL-2. These results confirm that the ID vaccination route induces CD8^+^ T-cell responses (48–50). Moreover, the IM route was the only one to induce a polyfunctional Th1-like CD4^+^ T-cell cluster. In contrast, the SC route induced transient increases of neutralizing antibodies and less polyfunctional CD4^+^ and CD8^+^ T cells. Aside from the polyfunctional T-cell phenotypic group, CD4^+^ and CD8^+^ T-cell cytotoxic; proliferative, and migrating phenotypic families were also affected by the administration route of MVA to a lesser extent, amplifying the dichotomy between the SC and ID/IM routes.

Among those families, the unsupervised analysis on CD4+ T cells highlighted the presence of CD4+ harboring a cytotoxic phenotype producing perforin and granzyme B. This cell population has been reported as a double edged sword able to either positively or negatively impact pathogenicity (51). In vaccination context, poliovirus specific cytotoxic CD4+ T have been described to efficiently kill *in vitro* infected cells (52). In line with results of this study, Munier et al. also highlighted a prominent increase of cytotoxic effector CD4+ T phenotype early after vaccinia virus vaccination (53).

Among the factors that could explain the specificity of the SC route relative to the ID and IM routes are the tissue composition and immune properties of the injection site. Indeed, subcutaneous tissue is dedicated to triglyceride storage and metabolic functions and is therefore mostly composed of adipocytes, with only a few macrophages and lymphocytes (54, 55). Nevertheless, the unique microvascularization of adipose tissue, which could facilitate the recruitment of cells from the blood, may amplify the magnitude and persistence of inflammation (56). In contrast, skin hosts a large diversity of cells dedicated to immune functions that are abundant at steady state (57). This could result in a better capacity to rapidly resolve the local inflammation induced by MVA (58) or conversely augment Ag presentation and the initiation of adaptive immunity. Normal muscle tissue contains only a few resident immune cells. However, IM injection of vaccines leads to significant recruitment of immune cells, creating local inflammation (59, 60). Another factor linking the immunization site to the immune outcome could be the capacity of MVA to infect local cells or be rapidly driven to the draining lymph node. Indeed, after ID injection, we observed E3L (an early expressed MVA protein) in the dermis but we did not detected its presence in the SC tissue after SC immunization (**Figure S4**), suggesting that tissue diffusion is faster after SC than ID administration of MVA. Although MVA does not require any specific receptor for infection (61), MVA-infected cells in the dermis are more abundant than in the SC tissue. This could lead to an increase in the local production of inflammatory mediators after ID injection (62). Furthermore, our results suggest that the time to reach the draining lymph node is dependent on the route of administration. Indeed, more abundant levels of E3L were detected in the subcapsular area of draining lymph nodes after SC injection, in accordance with literature on murine models (63, 64), whereas it was moderate after ID injection (**Figure S4**). Thus, the antigen-presenting cells that are mobilized might differ depending on the administration route. The persistence of the antigen in the skin after ID immunization suggests the possible uptake by local DC subsets, such as Langerhans cells or dermal DCs (57, 65).

Despite the data integration, this study did not identify key immune parameters that triggered the difference between the administration routes. Several relevant immune parameters were not included in this study such as NKT cells or innate lymphoid cells due to the design of the gating strategies. In addition, no direct correlation between innate and adaptive response could have been established because separate animal cohorts were used for each type of response due to injection site sampling requirements. The MVA was selected for this project as a model of live attenuated vaccine known to be highly immunogenic in addition to induce strong innate immune response. Other vaccine types such as inactivated, protein, polysaccharides or DNA/RNA are susceptible to use different pathways to activate the immune system. In this regard, further studies are necessary to extrapolate the impact of administration route to other vaccine responses.

Overall, we demonstrate that the administration route affects not only the magnitude but also the quality of the adaptive response to the MVA vaccine model. This modification of the adaptive response profile was probably shaped during the very early innate immune response. This work is in line with other studies highlighting the importance of immunization route for vaccination. Indeed, a major decrease of specific T cell response after intranasal immunization vs intravenous has been reported after BCG vaccination (66). Concomitantly, a decrease of humoral response, CD8+ T cell response, and protection has also been described after intramuscular immunization in comparison with intravenous using an attenuated malaria vaccine PfSPZ (44). Fowlpox-HIV vaccine has also been shown to differentially activate cytokine production of lung or muscle innate lymphoid cells according to the administration route (45). As a consequence the route of administration must be better considered in vaccine trials.

Further investigation is also needed to better understand why a given administration route induces more suitable immunity relative to the others. Such understanding will require identification of the key elements that drive the relationship between innate and adaptive responses. The use of systems vaccinology approaches combined with mathematical modeling to identify predictive biomarkers of adaptive immunity appear essential to harness and take advantage of these immune processes.

## Data Availability Statement

The raw data supporting the conclusions of this article will be made available by the authors, without undue reservation.

## Ethics Statement

The animal study was reviewed and approved by French research Ministry (12-013 and 0201501281731916 (APAF1S#170).02).

## Author Contribution

Conceptualization: PR, YL, RG, and FM. Methodology: PR, ND-B, A-SB, DP, AC, RG, and FM. Validation: PR, RG, and FM. Formal analysis: PR, CJ, and NT. Investigation: PR, AR, CJ, LS, and HH. Resources: NT and HH. Writing—original draft: PR. Writing—review and editing: PR, NT, A-SB, DP, RG, FM, and CJ. Funding acquisition: YL and RG. Supervision: YL, RG, and FM. All authors contributed to the article and approved the submitted version.

## Funding

This work was supported by the IDMIT infrastructure and funded by the ANR *via* grant No ANR-11-INBS-0008. N.T. held a fellowship from the ANRS (France Recherche Nord&Sud Sida-HIV Hépatites). This work was also supported by the “Investissements d’Avenir” programs managed by the ANR under reference ANR-10-LABX-77-01 funding the Vaccine Research Institute (VRI), Créteil (ImMemory research program) and ANR-10-EQPX-02–01 funding the FlowCyTech facility (IDMIT, Fontenay-aux-Roses, France). Funds were also received from the European Commission: ADITEC, FP7-HEALTH-2011-280873; Transvac, EU H2020 GA 730964; EHVA, EU H2020 GA 681032.

## Conflict of Interest

The authors declare that the research was conducted in the absence of any commercial or financial relationships that could be construed as a potential conflict of interest.

## Supplementary Material

Supplemental information provided with this manuscript contains 5 additional figures and 3 tables entitled as below:

Supplementary Figure 1 Analysis pipeline of the CyTOF dataset.

Supplementary Figure 2 Overview of the kinetics of differentially enriched clusters.

Supplementary Figure 3 Gating strategies used in flow cytometry to study the early innate response in blood.

Supplementary Figure 4 Skin and lymph node tissue after MVA immunization by the SC or ID route

Supplementary Figure 5 Gating strategy for the flow cytometry of tissue samples.

Supplementary Table 1 Panel used for CyTOF staining

Supplementary Table 2 Panel used for FlowCytometry staining

Supplementary Table 3 Dataset of innate immune response
