# Supplementary material for: Vaccine Inoculation Route Modulates Early Immunity and Consequently Antigen-Specific Immune Response
Source: Front Immunol. 2021 Apr 20;12:645210. doi: 10.3389/fimmu.2021.645210 (PMC8093451; doi:10.3389/fimmu.2021.645210)
Supplement: Supplementary Figure 1 — Analysis pipeline of the CyTOF dataset. [file DataSheet_1.pdf]

Supplementary Material

Vaccine inoculation route modulates early immunity and consequently antigen-specifc immune response

Authors:

Pierre Rosenbaum[1,2],Nicolas Tchitchek[1,2],Candie Joly[1,2],André Rodriguez Pozo[1,2],Lev Stimmer[3,4],Sébastien Langlois[1,2],Hakim Hocini[2,5], Leslie Gosse[1,2],David Pejoski[1,2],Antonio Cosma[1,2],Anne-Sophie Beignon[1,2],Nathalie Dereuddre-Bosquet[1,2], Yves Levy[2,5], Roger Le Grand[1,2] and Frédéric Martinon[1,2,*]

Affiliations:

[1]CEA – Université Paris-Saclay – INSERM U1184, Immunology of viral infections and autoimmune diseases, IDMIT Department, IBFJ, 92265 Fontenay-aux-Roses, France

[2]Vaccine Research Institute, Henri Mondor Hospital, 94010 Créteil, France

[3]INSERM, U1169, 94270 Kremlin-Bicêtre, France

[4]CEA – INSERM, MIRCen, UMS27, 92265 Fontenay-aux-Roses, France

[5]INSERM, U955, Team 16, Clinical and Infectious Diseases Department, Hospital Henri Mondor, University of Paris East, 94010 Créteil, France

*__Corresponding author:__

Correspondence should be addressed to: Frédéric Martinon, CEA, 18 Route du Panorama, 92265 Fontenay-aux-Roses, France (frederic.martinon@cea.fr).

Supplementary Materials:

Fig. S1. Analysis pipeline of the CyTOF dataset.

Fig. S2. Overview of the kinetics of differentially enriched clusters.

Fig. S3. Gating strategies used in flow cytometry to study the early innate response in blood.

Fig. S4. Skin and lymph node tissue after MVA immunization by the SC or ID route

Fig. S5. Gating strategy for the flow cytometry of tissue samples.

Table S1: Panel used for CyTOF staining

Table S2: Panel used for FlowCytometry staining

Table S3: Dataset of innate immune response

Fig. S1. Analysis pipeline of the CyTOF dataset.

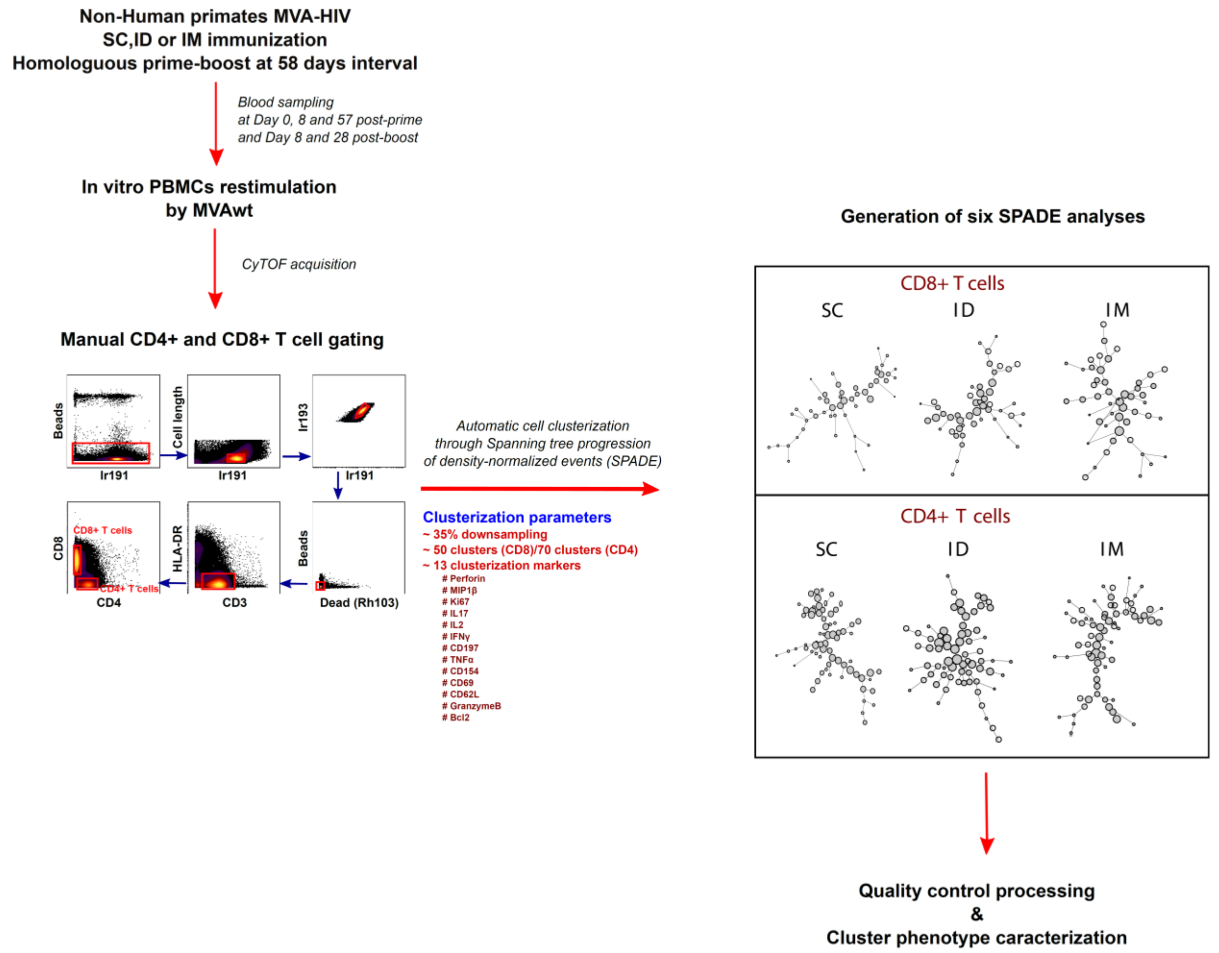

CyTOF analysis pipeline adapted from a previously developed method *(43)*. For the three SPADE representations, each node represents a cell cluster and its size is proportionnal to the total number of cells in the cluster, regardless of animal or timepoint. For circular representations, each dot at the extremity represents a cell cluster and the group assignations (polyfunctional, cytotoxic, proliferative, migrating, or others) correspond to those shown in Fig. S1 and 2. Red links correspond to significant correlations between clusters of the same group. Gray links correspond to significant correlations between clusters of different groups (student t-test, $p < 0.001$).

Fig. S2. Overview of the kinetics of differentially enriched clusters

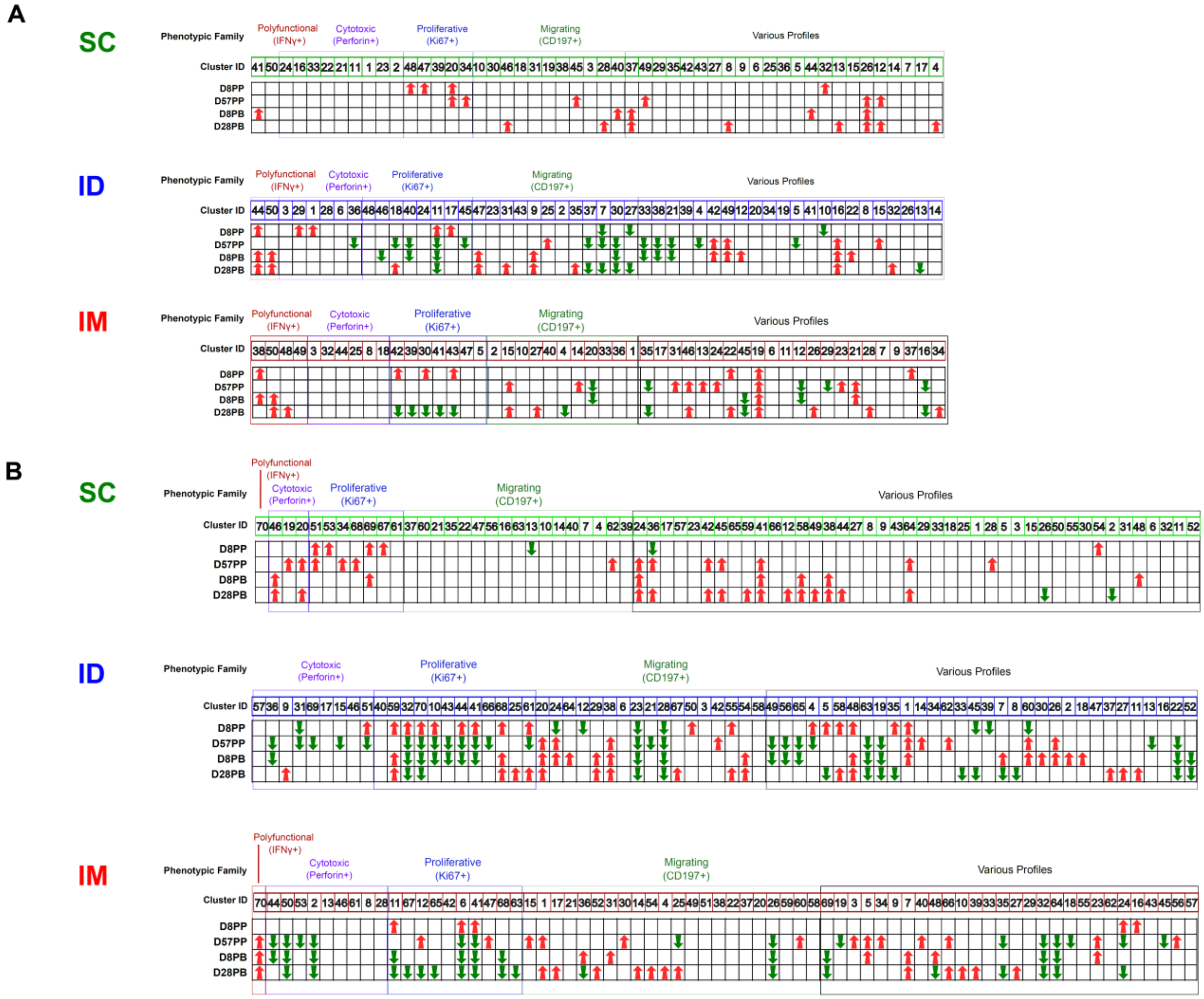

(A) CD8+ T-cell clusters. (**B**) CD4+ T-cell clusters. The upward-pointing red arrows indicate significant increases and the downward pointing green arrows depict significant decreases (t-test showing a p-value < 0.05, n = 4) in cell recruitment at the indicated timepoint relative to baseline.

Fig. S3. Gating strategies used in flow cytometry to study the early innate response in blood.

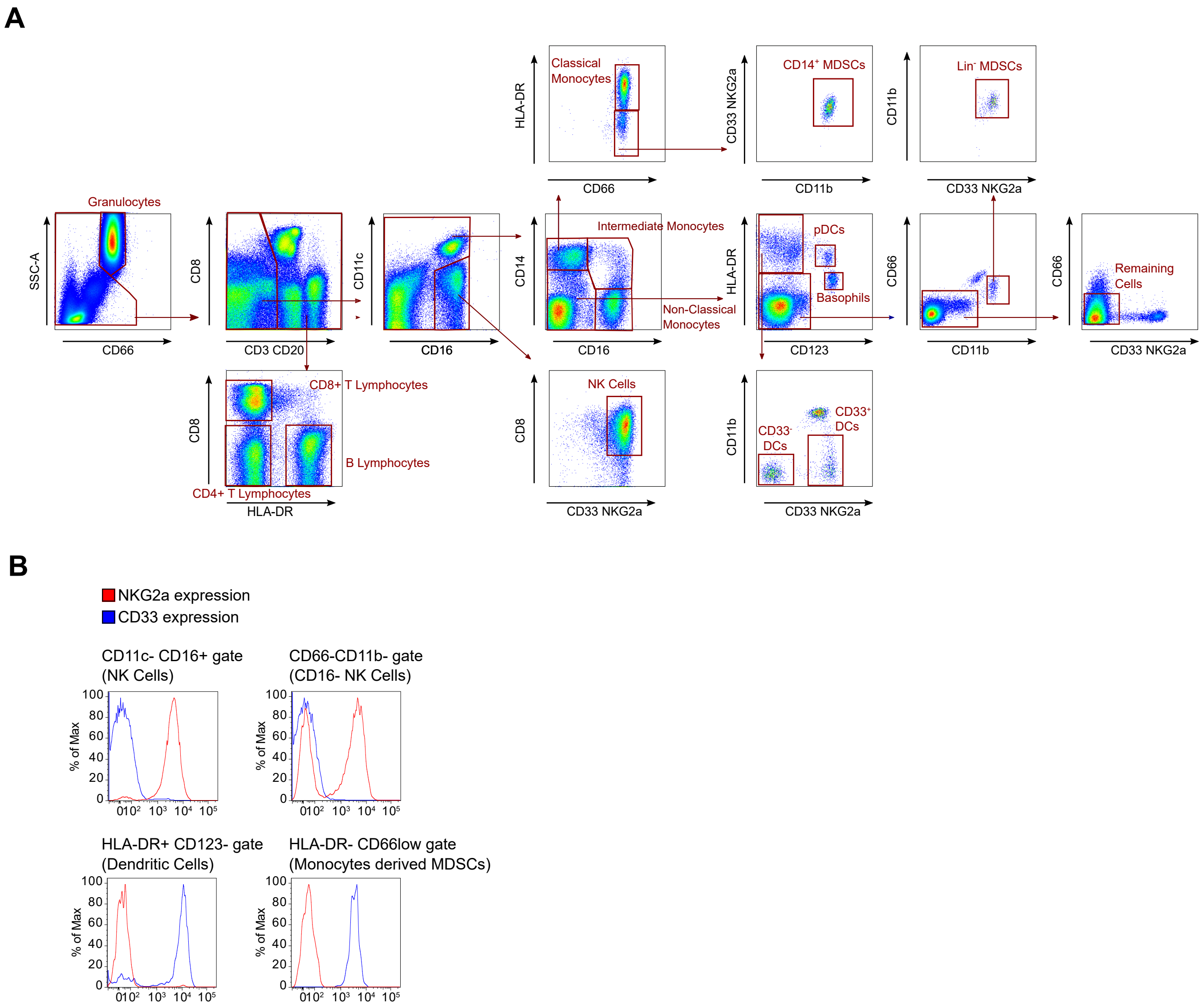

(**A**) Dot-plot representation of one representative experiment under conditions of inflammation. (**B**) Histograms showing the exclusivity of the NKG2a and CD33 markers, justifying the choice of using the same fluorochrome for both markers in the staining panel.

Fig. S4. Skin and lymph node tissue after MVA immunization by the SC or ID route.

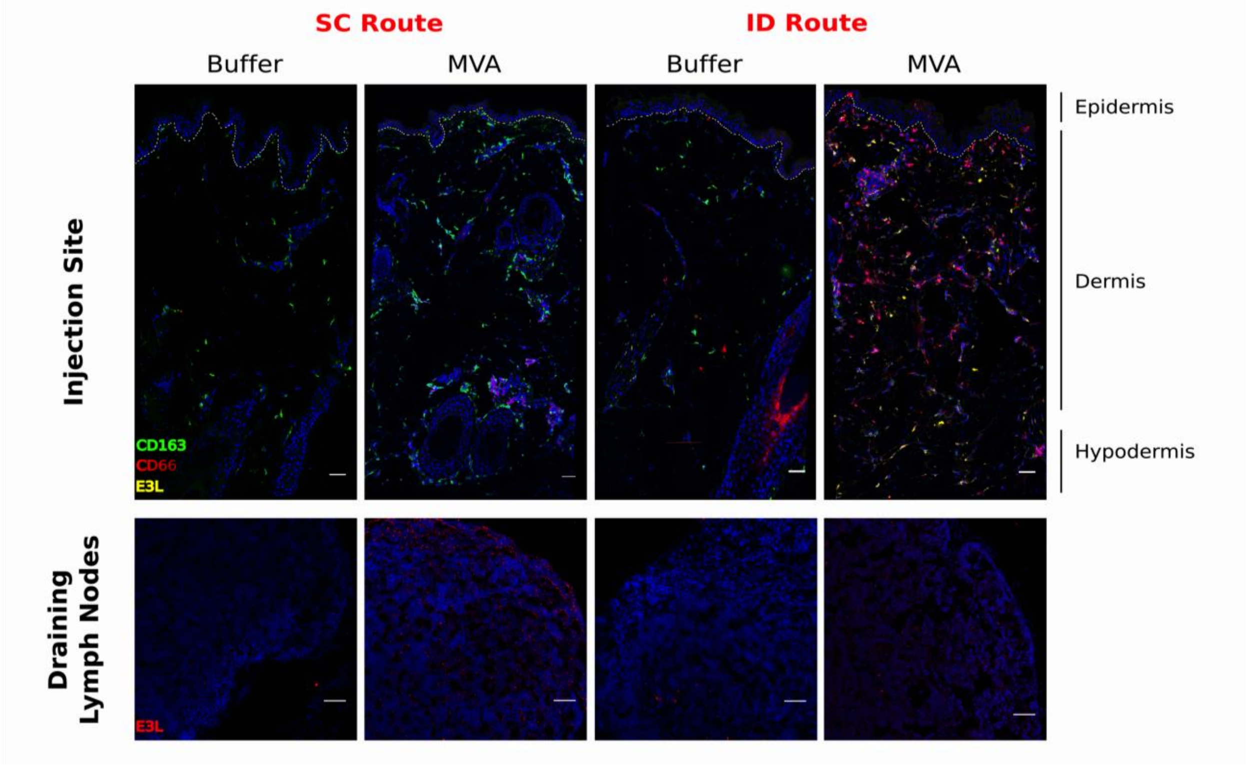

(**A**) Immunohistofluorescence of the injection site (skin and subcutaneous tissue) and draining inguinal lymph node, showing transversal sections of frozen cassettes 24 h after the injection of buffer or rMVA by the ID or SC route. The scale bar is equal to 50 µm. For the injection site: CD163 in green, CD66 in red, and E3L in yellow. The white dotted line represents the interface between epidermis and dermis. For the lymph nodes, E3L is in red. One representative experiment of two is shown.

Fig. S5. Gating strategy for the flow cytometry of tissue samples.

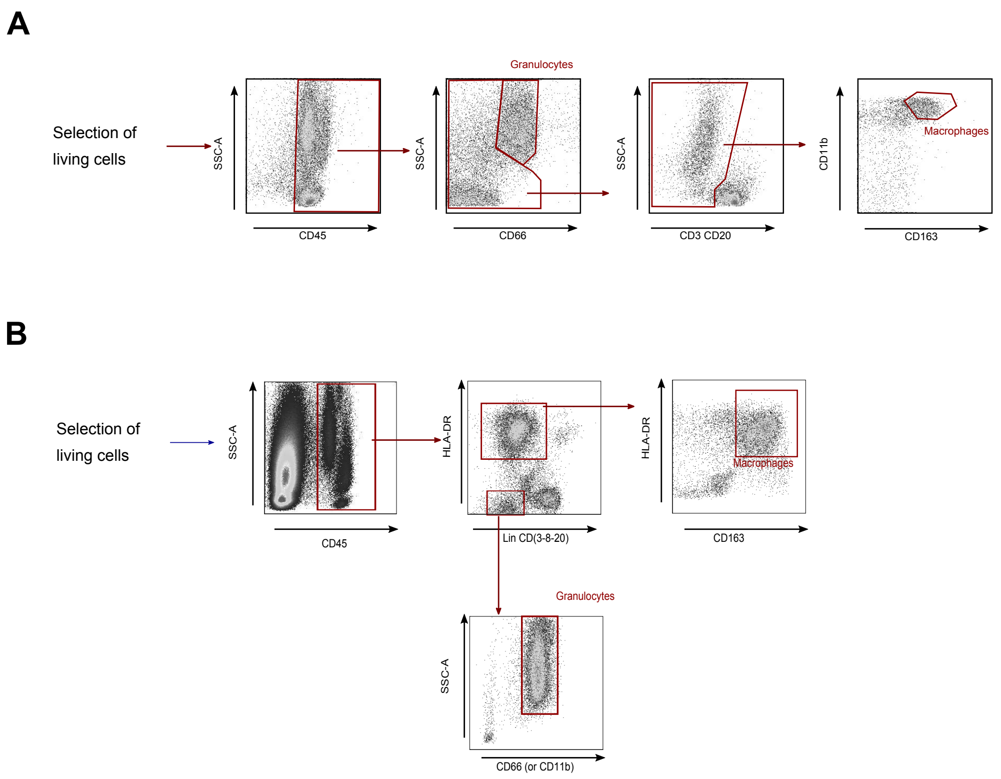

(**A**) Flow cytometry gating strategy for skin and subcutaneous cells. After doublet and debris exclusion, living cells were selected, and CD45 used to distinguish leukocytes from non-leukocytes. Among $CD45^+$ cells, granulocytes were first identified by their $CD66^+$ and $SSC\text{-}A^+$ phenotype. Among $CD45^+$ $CD66^-$ cells, lymphocytes were regrouped as one lineage $(CD3, CD20)^+$. Macrophages were identified as $CD45^+$, Lin $(CD3, CD20)^-$, $CD66^-$, $CD11b^+$, and $CD163^+$ cells. (**B**) Flow cytometry gating strategy for muscle cells. After doublet and debris exclusion, living cells were selected, and CD45 used to distinguish leukocytes from non-leukocytes. Among $CD45^+$ cells, $HLA\text{-}DR^+$ cells were first identified. Among $HLA\text{-}DR^+$ cells, macrophages were regrouped as $CD163^+$ cells. $HLA\text{-}DR^-$, lineage $(CD3, CD20)^-$ and $CD66^+$ or $CD11b^+$ cells were considered to be granulocytes.

Table S1. Panel used for CyTOF staining

Overview of markers used to identify the $CD4^{+}$ and $CD8^{+}$ T-cell profiles.

Metal	Antigen	Clone	Cytof optimal µl/well	Supplier(s)	Catalog
Nd(146)	HLA-DR	L243 (G46-6)	0,05	BD Biosciences	555810
Gd(156)	CD45RA	5H9	0,3	BD Biosciences	556625
Nd(150)	CD62L	SK11	0,3	BD Biosciences	559050
Dy(162)	CD279 (PD-1)	EH12.2H7	0,5	Biolegend	BLE329902
Yb(172)	CD95	DX2	1,3	BD Biosciences	555671
Er(168)	CD195 (CCR5)	3A9	1	BD Biosciences	556041
Nd(145)	CD197 (CCR7)	G043H7	1	Biolegend	BLE353202
Eu(151)	CD27	O323	1	Biolegend	BLE302802
Sm(147)	CD278 (ICOS)	C398.4A	2	Biolegend	BLE313502
Nd(144)	CD28	CD28.2	1,5	BD Biosciences	555726
Yb(174)	CD127 (IL-7R)	eBioRDR5	0,7	eBiosciences	16-1278-82
Sm(152)	CD185 (CXCR5)	710D82.1	0,7	NIH	710D82.1
Nd(148)	GranzymeB	GB11	0,05*	Clinisciences	C112623
Lu(175)	TNF-α	MAb11	0,5*	BD Biosciences	559071
Tm(169)	CD4	L200	0,2*	BD Biosciences	550625
Sm(154)	CD8	RPA-T8	0,1*	BD Biosciences	555364
Eu(153)	CD40L (CD154)	TRAP1	0,2*	BD Biosciences	555698
Gd(158)	IFN-γ	B27	0,1*	BD Biosciences	51-410-20661
Dy(164)	MIP-1β (CCL4)	D21-1351	0,1*	BD Biosciences	51-410-23851
Ho(165)	IL-2	MQ1-17H12	0,1*	BD Biosciences	51-410-18951

Yb(176)	IL-10	JES3-9D7	0,1*	Miltenyi Biotec	130-096-041
Pr(141)	IL-17	eBio64DEC 17	0,1*	eBiosciences	14-7179-82
Gd(160)	Ki-67	B56	0,2*	BD Biosciences	51-410-36521
Er(167)	IL-4	7A3-3	0,2*	Miltenyi Biotec	120-000-031
Nd(143)	CD3	SP34-2	0,3*	BD Biosciences	551916
Tb(159)	Bcl-2	Bcl-2/100	0,3*	eBiosciences	14-1028-82
Yb(171)	Perforin	Pf-344	0,2*	MabTech	3465-5-250
Nd(142)	CD69	FN50	0,1*	BD Biosciences	5555529
Sm(149)	FoxP3	206D	0,3*	Biolegend	320102
Ir(191/193)	DNA	N/A	1/500	DVS Sciences	201192A
Rh(103)	Viability-103	N/A	1/200	DVS Sciences	201103A

*Intracellular staining

Clustering Markers

Table S2. Panel used for Flow Cytometry staining.

Overview of markers used to discriminate between immune cells in the blood and tissues.

Staining	Marker	Fluorochrome	Clone
Blood	CD66abce	FITC	TET2
Blood	CD123	PerCP	7G3
Blood	CD11c	PE-Cy7	3.9
Blood	CD3	APCH7	SP34-2
Blood	CD20	APCH7	2H7
Blood	CD16	APC	3G8
Blood	CD11b	AF700	Bear 1
Blood	CD14	V450	M5E2
Blood	HLA-DR	V500	G46-6
Blood	CD8	BV650	RPA-T8
Blood	NKG2a	PE	Z199
Blood	CD33	PE	AC104.3E3
Skin and subcutaneous cell suspension	CD45	PerCP-Cy5.5	DO58-1283
Skin and subcutaneous cell suspension	CD66abce	APC	TET2
Skin and subcutaneous cell suspension	CD163	BV711	GHI/61
Skin and subcutaneous cell suspension	CD3	V450	SP34-2
Skin and subcutaneous cell suspension	CD20	V450	2H7
Skin and subcutaneous cell suspension	CD16	APC	3G8
Skin and subcutaneous cell suspension	CD11b	PE-Cy7	Bear 1
Muscle cell suspension	CD45	PerCP	DO58-1283
Muscle cell suspension	HLA-DR	APC-H7	G46-6
Muscle cell suspension	CD3	V450	SP34-2
Muscle cell suspension	CD8	V450	RPA-T8
Muscle cell suspension	CD20	V450	2H7
Muscle cell suspension	CD66abce	FITC	TET2
Muscle cell suspension	CD11b	PE-Cy7	Bear 1
Muscle cell suspension	CD163	AF700 (Zenon labelling kit (Life))	GHI/61

Table S3. Dataset of innate immune response.

(File sent in separately)

Overview of innate parameters used to perform the integrative analyses. Primary data for each cell population are expressed in percentage among $CD45^+$ cells. Cell populations and gene expression are then expressed as the ratio between value at 24h after MVA injection and untreated condition.
