# Supplementary material for: Vaccine Inoculation Route Modulates Early Immunity and Consequently Antigen-Specific Immune Response
Source: Front Immunol. 2021 Apr 20;12:645210. doi: 10.3389/fimmu.2021.645210 (PMC8093451; doi:10.3389/fimmu.2021.645210)
Supplement: Supplementary Table 1 — Panel used for CyTOF staining [file Table_1.docx]

**KEY RESOURCES TABLE**

| REAGENT or RESOURCE | SOURCE | IDENTIFIER |
| --- | --- | --- |
| Antibodies | | |
| Anti-HLA-DR purified (clone L243) | BD Biosciences | Cat# 555810 |
| Anti-CD45RA purified (clone 5H9) | BD Biosciences | Cat# 556625 |
| Anti-CD62L purified (clone SK11) | BD Biosciences | Cat# 559050 |
| Anti-CD279 purified (clone EH12.2H7) | Biolegend | Cat# BLE329902 |
| Anti-CD95 purified (clone DX2) | BD Biosciences | Cat# 555671 |
| Anti-CD195 purified (clone 3A9) | BD Biosciences | Cat# 556041 |
| Anti-CCR7 purified (clone G043H7) | Biolegend | Cat# BLE353202 |
| Anti-CD27 purified (clone O323) | Biolegend | Cat# BLE302802 |
| Anti-CD278 purified (clone C398.4A) | Biolegend | Cat# BLE313502 |
| Anti-CD28 purified (clone CD28.2) | BD Biosciences | Cat# 555726 |
| Anti-CD127 purified (clone eBioRDR5) | eBiosciences | Cat# 16.1278‑82 |
| Anti-CD185 purified (clone 710D82.1) | NIH | Cat# 710D82.1 |
| Anti-GranzymeB purified (clone GB11) | Clinisciences | Cat# C112623 |
| Anti-TNFα purified (clone Mab11) | BD Biosciences | Cat# 559071 |
| Anti-CD4 purified (clone L200) | BD Biosciences | Cat# 550625 |
| Anti-CD8 purified (clone RPA‑T8) | BD Biosciences | Cat# 555364 |
| Anti-CD40L purified (clone TRAP1) | BD Biosciences | Cat# 555698 |
| Anti-IFNγ purified (clone B27) | BD Biosciences | Cat# 51‑410‑36521 |
| Anti-MIP1β purified (clone D21-1351) | BD Biosciences | Cat# 51‑410‑23851 |
| Anti-IL2 purified (clone MQ1-17H12) | BD Biosciences | Cat# 51‑410‑18951 |
| Anti-IL10 purified (clone JES3-9D7) | Miltenyi Biotec | Cat# 130‑096‑041 |
| Anti-IL17 purified (clone eBio64DEC17) | eBiosciences | Cat# 14‑7179‑82 |
| Anti-Ki67 purified (clone B56) | BD Biosciences | Cat# 51-410-36521 |
| Anti-IL4 purified (clone 7A3.3) | Miltenyi Biotec | Cat# 120-000-031 |
| Anti-CD3 purified (clone SP34‑2) | BD Biosciences | Cat# 551916 |
| Anti-BCl2 purified (clone Bcl‑2/100) | eBiosciences | Cat# 14-1028-82 |
| Anti-Perforin purified (clone Pf‑344) | MabTech | Cat# 3465-5-250 |
| Anti-CD69 purified (clone FN50) | BD Biosciences | Cat# 5555529 |
| Anti-FoxP3 purified (clone 206D) | Biolegend | Cat# 320102 |
| Anti‑CD66abce FITC (clone TET2) | Miltenyi Biotec | Cat# 130-093-132 |
| Anti‑CD123 PerCP (clone 7G3) | BD Biosciences | Cat# 558714 |
| Anti‑CD11c PE‑Cy7 (clone 3.9) | Biolegend | Cat# 301608 |
| Anti‑CD3 APC‑H7 (clone SP34‑2) | BD Biosciences | Cat# 557757 |
| Anti‑CD20 APC‑H7 (clone 2H7) | BD Biosciences | Cat# 560853 |
| Anti‑CD16 APC (clone 3G8) | Miltenyi Biotec | Cat# 302012 |
| Anti‑CD11b AF700 (clone Bear1) | Beckman Coulter | Cat# IM3611 |
| Anti‑CD14 V450 (clone M5E2) | BD Biosciences | Cat# 561390 |
| Anti‑HLA‑DR V500 (clone G46‑6) | BD Biosciences | Cat# 561224 |
| Anti‑CD8 BV650 (clone RPA‑T8) | BD Biosciences | Cat# 563822 |
| Anti‑NKG2a PE (clone Z199) | Beckman Coulter | Cat# IM3291U |
| Anti‑CD33 PE (clone AC104.3E3) | Miltenyi Biotec | Cat# 130-113-911 |
| Anti‑CD45 PerCP-Cy5.5 (clone  DO58-1283) | BD Biosciences | Cat# 558411 |
| Anti‑CD66abce APC (clone TET2) | Miltenyi Biotec | Cat# 130-118-539 |
| Anti‑CD163 BV711 (clone GHI/61) | BD Biosciences | Cat# 563889 |
| Anti‑CD11b PE‑Cy7 (clone Bear1) | Beckman Coulter | Cat# A54822 |
| Anti‑CD8 V450 (clone RPA‑T8) | BD Biosciences | Cat# 561426 |
| Anti‑CD163 purified (clone GHI/61) | Biolegend | Cat# 333602 |
| Goat anti Monkey IgG (H/L) | BioRad | Cat# AAI42 |
|  |  |  |
| Bacterial and Virus Strains | | |
| MVAwt | Transgene | N/A |
| MVA-HIVB | Transgene | N/A |
| MVA-eGFP | Transgene | N/A |
|  |  |  |
| Biological Samples |  |  |
| Macaca Fascicularis | N/A | N/A |
|  |  |  |
|  |  |  |
|  |  |  |
| Chemicals, Peptides, and Recombinant Proteins | | |
| Zenon labeling kit | Thermofischer Scientific | Z25011 |
| LIVE/DEAD™ Fixable Blue Dead Cell Stain Kit | Fischer Scientific | L23105 |
|  |  |  |
| Critical Commercial Assays | | |
| Microarray chips | Illumina | Human HT-12 v4.0 |
|  |  |  |
| Deposited Data | | |
|  | Array Xpress | E-MTAB-10309 |
| Software and Algorithms | | |
| GraphPad Prism | GraphPad | N/A |
| FlowJo | Tree Star | N/A |
| R packages (Cytocompare, SpadeVizR) | N/A | N/A |
|  |  |  |
|  |  |  |
|  |  |  |
